# Structure-Based Identification of Natural-Product-Derived Compounds with Potential to Inhibit HIV-1 Entry

**DOI:** 10.3390/molecules28020474

**Published:** 2023-01-04

**Authors:** Nneka Ugwu-Korie, Osbourne Quaye, Edward Wright, Sylvester Languon, Odame Agyapong, Emmanuel Broni, Yash Gupta, Prakasha Kempaiah, Samuel K. Kwofie

**Affiliations:** 1 West African Centre for Cell Biology of Infectious Pathogens, Department of Biochemistry, Cell and Molecular Biology, College of Basic and Applied Sciences, University of Ghana, Legon, Accra P.O. Box LG 54, Ghana; 2 School of Life Sciences, University of Sussex, Brighton BN1 9QG, UK; 3 Cellular and Molecular Biomedical Sciences Program, University of Vermont, Burlington, VT 05405, USA; 4 Department of Biomedical Engineering, School of Engineering Sciences, College of Basic & Applied Sciences, University of Ghana, Legon, Accra P.O. Box LG 77, Ghana; 5 Department of Parasitology, Noguchi Memorial Institute for Medical Research (NMIMR), College of Health Sciences (CHS), University of Ghana, Legon, Accra P.O. Box LG 581, Ghana; 6 Department of Medicine, Loyola University Medical Center, Maywood, IL 60153, USA; 7 Infectious Diseases, Mayo Clinic, Jacksonville, FL 32224, USA

**Keywords:** HIV-1 entry, inhibition, CD4-binding site, VRC01, gp120, virtual screening, small compound mimetics

## Abstract

Broadly neutralizing antibodies (bNAbs) are potent in neutralizing a wide range of HIV strains. VRC01 is a CD4-binding-site (CD4-bs) class of bNAbs that binds to the conserved CD4-binding region of HIV-1 envelope (env) protein. Natural products that mimic VRC01 bNAbs by interacting with the conserved CD4-binding regions may serve as a new generation of HIV-1 entry inhibitors by being broadly reactive and potently neutralizing. This study aimed to identify compounds that mimic VRC01 by interacting with the CD4-bs of HIV-1 gp120 and thereby inhibiting viral entry into target cells. Libraries of purchasable natural products were virtually screened against clade A/E recombinant 93TH057 (PDB: 3NGB) and clade B (PDB ID: 3J70) HIV-1 env protein. Protein–ligand interaction profiling from molecular docking and dynamics simulations showed that the compounds had intermolecular hydrogen and hydrophobic interactions with conserved amino acid residues on the CD4-binding site of recombinant clade A/E and clade B HIV-1 gp120. Four potential lead compounds, NP-005114, NP-008297, NP-007422, and NP-007382, were used for cell-based antiviral infectivity inhibition assay using clade B (HXB2) env pseudotype virus (PV). The four compounds inhibited the entry of HIV HXB2 pseudotype viruses into target cells at 50% inhibitory concentrations (IC_50_) of 15.2 µM (9.7 µg/mL), 10.1 µM (7.5 µg/mL), 16.2 µM (12.7 µg/mL), and 21.6 µM (12.9 µg/mL), respectively. The interaction of these compounds with critical residues of the CD4-binding site of more than one clade of HIV gp120 and inhibition of HIV-1 entry into the target cell demonstrate the possibility of a new class of HIV entry inhibitors.

## 1. Introduction

Human immunodeficiency virus (HIV) entry is a complex interaction of host cell CD4 receptor with chemokine co-receptors (CXCR4 and CCR5) and the HIV env protein (trimer of gp120 non-covalently bound to gp41). The process comprises attachment to the host cell, binding of CD4 receptor, co-receptor binding, cell fusion, and deposition of viral genetic material for viral replication [1]. Targeting HIV entry into the host cell presents opportunities for therapeutic intervention as a treatment and prevention strategy for viral infection [2,3]. Only two viral entry inhibitors (enfuvirtide (fusion inhibitor) and maraviroc (CCR5 antagonist)) have been approved for use in the clinical treatment of HIV by the US Food and Drug Administration (FDA) [2]. However, there are limitations associated with the clinical use of these entry inhibitors for the treatment of HIV infection. Treatment with maraviroc is associated with the emergence of CXCR4 tropic viruses [4,5], and enfuvirtide is a large polypeptide administered subcutaneously which is associated with painful injection sites and therefore limits the clinical use of the drug [6]. 

Broadly neutralizing antibodies (bNAbs) of human immunodeficiency type-1 (HIV-1) are monoclonal antibodies that solely target the env protein of HIV-1. They possess exceptional potency and breadth against variant strains of HIV-1 due to their ability to interact with conserved regions of HIV env protein [7,8,9]. These bNAbs have been shown to completely prevent simian–human immunodeficiency virus (SHIV) infections [10], suppress viremia in HIV-1-infected individuals [11], and effectively control HIV-1 infection and suppress viremia in humanized mice after therapy was discontinued for about sixty days—owing to the longer half-life of the antibodies compared to antiretrovirals (ARVs) [12,13,14].

CD4-binding-site (CD4bs) bNAbs exhibit high potency and breadth to variant HIV strains, since the binding site is one of the most functionally conserved sites of the HIV-1 env protein and is required for initial CD4 binding and successful infection of a host cell by HIV [15]. VRC01 is an example of CD4bs bNAbs with a neutralization breadth of about 80–90% of HIV strains and a high potency (IC_50_ of 0.33 µg/mL) against HIV group M [8] and clade B HIV-1 (IC_50_ between 10 and 25 µg/mL in a TZM-bl neutralization assay) [16]. As a CD4-bs, VRC01 shares certain similarities with CD4. The VRC01 binding site on HIV-1 gp120 is 98% similar to that of CD4 [17], although CD4 prefers gp120 in an unbound state, while VRC01 has an affinity for gp120 in both CD4-bound and unbound states [17]. Other similarities between CD4 and VRC01 include the hydrogen bonding interaction between Arg59_CD4_ and Gly54_VRC01_ with Asp368_gp120_ [17]. VRC01 has demonstrated efficacy in the prevention of HIV in humanized mice, prolonged the time of HIV rebound during treatment interruption, and is currently in clinical trials for prevention of HIV transmissions in human [18,19,20]. Furthermore, VRC01 infusions into chronically HIV-1-infected individuals showed that VRC01 temporarily prevented viral replication in study participants, implying that at higher VRC01 concentrations, long-term viral rebound regulation could be achieved [21].

Even though HIV bNAbs such as VRC01 have the potential of being broad and potent HIV entry inhibitors due to promising results as a potential therapeutic agent, the antibodies are saddled with challenges that are associated with development as therapeutics. The challenges include laborious and time-consuming production techniques, high cost of production, instability of the developed antibody, delivery systems of the antibodies, and risk of treatment failure due to variation in immunological responses of antibodies therapy [22]. Moreover, the different clades have mutations in gp120 protein affecting the neutralization potential of these antibodies [23]. To overcome these challenges, small molecule mimetics of large biological molecules can be developed as therapeutic agents against HIV infection.

Natural products (NP) and their derivatives have traditionally played an important role in drug discovery [24]. Libraries of NPs and their derivatives are distinguished by the complexity and diversity of their structures in addition to their molecular rigidity when compared to the synthetic small compound libraries [25]. These characteristics confer an advantage when inhibiting protein–protein interactions [26]. Compounds derived from NPs have been shown to inhibit critical steps in HIV infections [27,28]. Arylnaphthaline lignin glycoside, “patentiflorin A”, which was derived from the *Justica gendarussa* plant, showed inhibitory activity against HIV isolates resistant to azidothymidine (AZT) and nevirapine (NVP). Calanolide A and B derived from the mangosteen family tree *Calophyllum lanigerum* have exhibited inhibition of non-nucleoside reverse transcriptase (NNRT) and are currently in development. Furthermore, *Rheum palmatum L*, Kuwanon-L, and Betulinic acid have demonstrated HIV integrase inhibition [27].

In silico techniques have been used in the early stages of drug discovery to identify hit compounds and elucidate the mechanism of action of natural products, thereby lowering time and costs and increasing efficiency [29,30]. Previous studies have combined cheminformatics-based and experimental-based studies to identify inhibitors for various diseases [31,32]. Computational approaches such as molecular docking, high-throughput virtual screening, molecular dynamics simulations, and quantitative structure–activity relationship (QSAR) have also been deployed in HIV research to discover small compounds with potential activity against HIV [33,34,35,36].

This study, therefore, sought to use a computational approach to identify compounds that mimic the broadly neutralizing antibody VRC01 in interaction with conserved amino acid residues of CD4-bs and assess the in vitro inhibitory activity of these mimetic compounds on HIV-1 infection using pseudotype technology.

## 2. Results

### 2.1. In Silico High-Throughput Screening of Natural Compound Mimetics of VRC01

#### 2.1.1. Protein Structure Extraction

The crystal structure of PDB ID: 3NGB with a resolution of 2.68 Å was obtained from the Protein Data Bank (PDB). 3NGB is a complex of the crystallized antigen-binding fragment (Fab) of the broadly neutralizing antibody VRC01 and the core gp120 of HIV-1 clade A/E recombinant 93TH057. The protein consists of twelve chains comprising G, A, D, I, H, B, E, J, L, C, F, and K. Chains G, A, D, and I are HIV-1 envelope glycoprotein, chains H, E, J, and B are the heavy chains of VRC01 Fab, while chains L, C, F, and K are the light chains of VRC01 Fab. The core gp120 trimer (chains G, A, and D) consists of outer and inner domain with truncated N- and C-termini and V1/V2 and V3 variable loops [17,37].

#### 2.1.2. Determination of gp120 Core Residues of Interaction with VRC01

**Figure 1:**
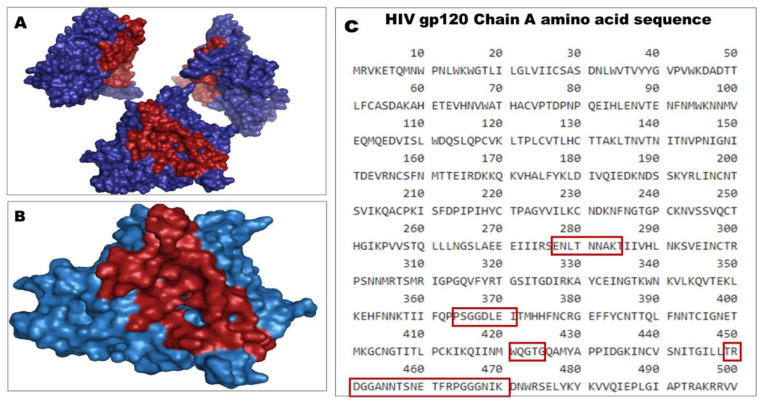
HIV-1 gp120 site of vulnerability for VRC01. (**A**) gp120 trimer (3J70; HXB2) with the site of vulnerability (red), (**B**) gp120 monomer (chain A) with the site of vulnerability (red), and (**C**) gp120 amino acid sequence (chain A) with residues of interaction with VRCO1 (red boxes).

The site of vulnerability on the gp120 site is the highly conserved contact site for the CD4 receptor on the gp120 outer domain. VRC01 contacts amino acid residues of gp120 at positions 275–283, 364–371, 421–437, and 455–478, which represent 98% of the CD4-binding site on the HIV-1 gp120 (Figure 1).

#### 2.1.3. Molecular Dynamics (MD) Simulation 

**Figure 2:**
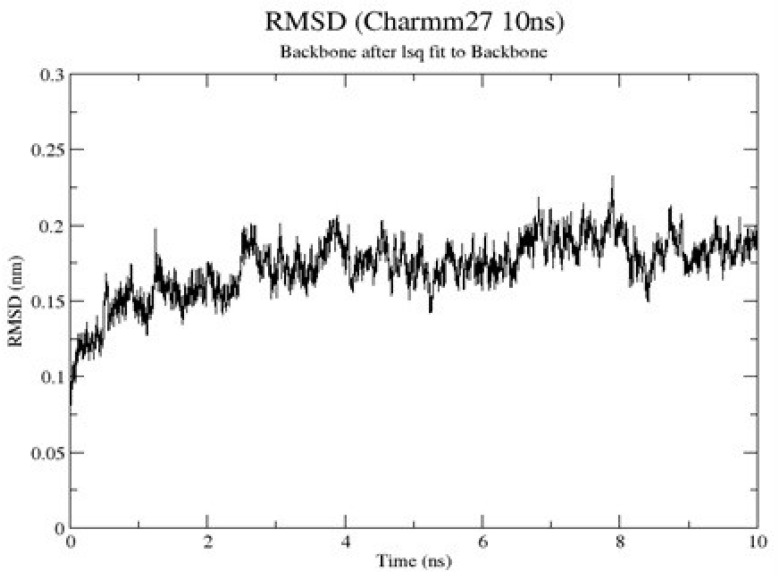
Root-mean-square deviation (RMSD) of the MD simulation of HIV-1 gp120 monomer using GROMACS. A plot of RMSD in nanometers (nm) against time in nanoseconds (ns). The RMSD increased from 0 ns to 1 ns and fluctuated till 9 ns, after which it stabilized till the end of the simulation.

The results obtained from the MD simulation of the receptor indicated that the root-mean-square deviation (RMSD) of the protein backbone steadily increased from 0.075 nm at 0 ns to 0.175 nm at 1 ns. There was a constant fluctuation of the RMSD between 0.15 nm (lowest point) and slightly above 0.225 nm (highest point) within the period of 1 ns and 9 ns (Figure 2). The system stabilized after 9 ns between about 0.175 nm and 0.2 nm till the end of the simulation. The low RMSD after 9 ns is indicative of fewer fluctuations and a more stable protein structure [38].

#### 2.1.4. Ligand Generation, Preparation, and Molecular Docking

Six purchasable natural product-derived compound libraries were downloaded from three natural compound companies. A total of 27,824 distinct ligands were generated from these compound libraries (Table 1). The ligands were minimized and optimized using the default setting in OpenBabel via PyRx. A total of 27,824 ligands generated from six natural product libraries (Table 1) were used for molecular docking using AutoDock Vina. The AutoDock molecular docking program assumes a rigid receptor and a conformationally flexible ligand. Different conformational poses of the ligands were docked into a rigid receptor (HIV gp120) and scored according to the binding energies of the receptor–ligand complexes. The best ligand poses with the lowest binding energies were selected and visualized in PyMOL.

#### 2.1.5. Protein–Ligand Interaction Profiling

**Figure 3:**
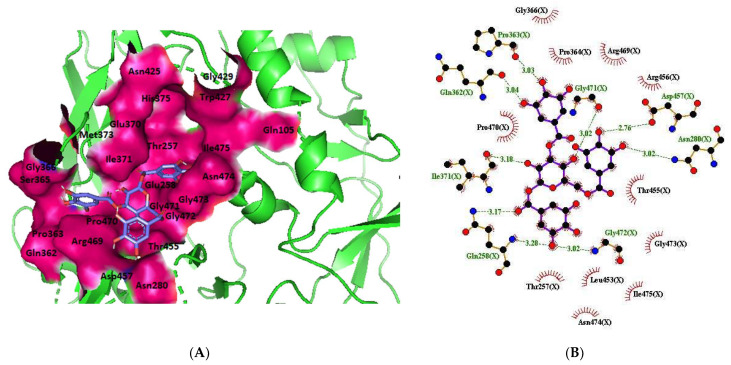
HIV gp120 of recombinant clade A/E-NP-005114 complex interaction profile. (**A**) Molecular representation of HIV gp120 of recombinant clade A/E in complex with compound NP-005114 (represented as sticks, firmly docked into CD4-bs (colored hot pink) of HIV gp120). (**B**) Protein–ligand interaction profile evaluated using LIGPLOT. In (**B**), the green dotted lines represent hydrogen bond interactions, while red arcs with spikes represent hydrophobic interactions. Residues of the receptor are shown as orange lines with black and red dots, while the ligand is shown as purple lines with black and red dots. Ligand NP-005114 had 9 hydrogen bonds and 11 hydrophobic bonds with residues in the CD4-bs of HIV gp120.

LIGPLOT was used to elucidate the amino acid residues in the binding site of the receptor (HIV gp120) that interact with the ligands [39], and 2D schematic diagrams that show the protein–ligand interactions were obtained. Ligand NP-005114 had nine hydrogen bond interactions with eight amino acid residues and hydrophobic interactions with eleven amino acid residues in the CD4-bs of HIV gp120 (Figure 3A,B). Ligand NP-008297 had ten hydrogen bond interactions with amino acid residues and hydrophobic interactions with eleven amino acid residues in the CD4-bs of HIV gp120 (Figures S3A and S4A). Ligand NP-007382 formed hydrogen bond interactions with five and seven hydrophobic interactions with amino acid residues in the CD4-bs (Figures S3B and S4B). The binding energies and interacting amino acid residues are summarized in Table 2.

#### 2.1.6. High-Throughput Virtual Screening (vHTS) and Analysis

The top 100 ligands from each of the six libraries (total 600) ranked according to their binding energies were analyzed for binding interactions with HIV-1 gp120. Out of a total of 470 ligands with high binding affinity analyzed from the selected 600 ligands, 386 ligands had hydrogen bond interactions with HIV gp120, while 84 ligands had no hydrogen bond interactions with HIV gp120. Of the 386 ligands with hydrogen bond interactions with gp120, 263 ligands had hydrogen bond interactions with less than four conserved amino acid residues within the site of vulnerability, while 41 had hydrogen bond interactions with four or more amino acid residues in the site of vulnerability on the HIV-1 gp120 (Figure S5). The best nine compounds were selected for in vitro evaluations based on their binding energies and the number of amino acid residues interacting with the HIV-1 gp120 CD4-binding site. The molecular structures of the nine selected compounds are shown in (Table 3). The binding energies of the best nine compounds ranged from −10.3 to −6.4 kcal/mol (Table 2). The lower value of the binding energies corresponds with a higher binding affinity to HIV-1 gp120.

#### 2.1.7. Determination of HIV HXB2 Envelope Protein Structure

Human immunodeficiency virus clade B clone 2 (HIV HXB2) served as the reference HIV strain. HXB2 envelope protein was used in the production of HIV-1 PVs for use in cell-based viral infectivity inhibition assays. The cDNA sequence of the HXB2 envelope protein (gp160) was retrieved from the HIV sequence database (Figure S6A) and converted into an amino acid sequence using the ExPASy Translate tool [40]. The HXB2 gp160 amino acid sequence was used to query the ExPASy server platform. The protein sequence (UniProtKB ID: P04578) with the highest similarity score to our query protein sequence was retrieved from UniProtKB (Figure S6B). The 3D structure of the protein (PDB ID: 3J70), with 100% structural similarity to P04578, was retrieved from Protein Data Bank. The structure consists of HIV HXB2 gp160 (chains D, P, U, E, Q, and V) in complex with CD4 (chains C, O, and T) and 17b antibody (chains A, M, R, B, N, and S) (Figure S6C). The HIV HXB2 gp120 trimer (chains D, P, and U) was extracted from 3J70 using PyMOL (Figure S6D).

#### 2.1.8. Virtual Screening and Analysis of HIV HXB2 Envelope Protein

**Figure 4:**
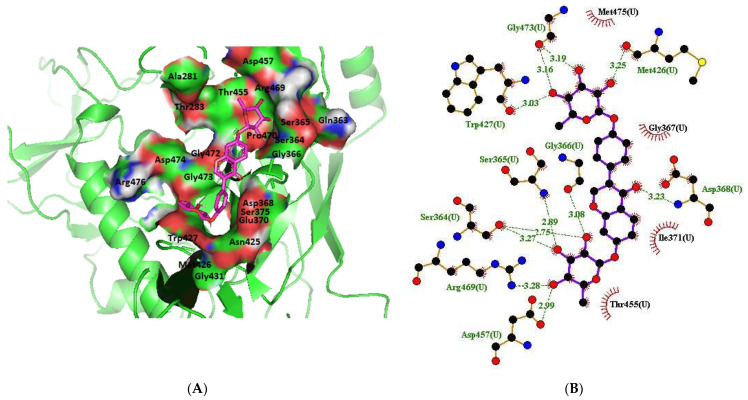
HIV gp120 of clade B-NP-005114 complex interaction profile. (**A**) Molecular representation of HIV gp120 of clade B in complex with compound NP-005114 (represented as sticks, firmly docked into CD4-bs of HIV gp120). (**B**) Protein–ligand interaction profile of NP-005114 and clade B gp120. NP-005114 had eleven hydrogen bond interactions and four hydrophobic interactions with amino acids in the CD4-bs.

The nine selected compounds were used for virtual screening on HIV-1 clade B envelope protein. The compounds were docked against the HXB2 gp120 monomer and interacted with the conserved CD4-bs on HXB2 gp120 (Figure 4A). NP-005114 had hydrogen bond interactions with eleven amino acids and hydrophobic interactions with four amino acids on the CD4-bs (Figure 4B). There were four and ten hydrogen and hydrophobic interactions, respectively, between NP-008297 (Figure S7A,B) and the amino acids in the gp120 CD4-bs. Compound NP-007382 had five hydrogen bond interactions and twelve hydrophobic interactions with the amino acids of CD4-bs (Figure S7C,D).

#### 2.1.9. RMSD of Clade A/E-Ligand Complexes

**Figure 5:**
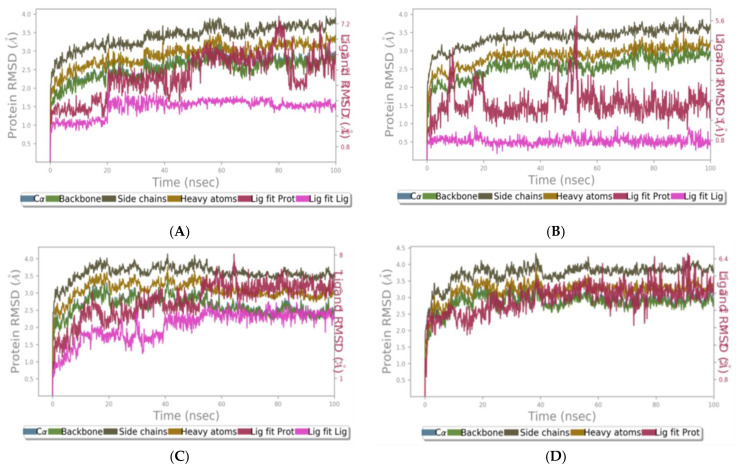
RMSD analysis of 100 ns MD simulation trajectory. The RMSD plots for clade A/E- (**A**) NP-005114, (**B**) NP-007382, (**C**) NP-007422, and (**D**) NP-008297 complexes.

RMSD was used to assess the binding stability of the complexes. For all the complexes, the C-alpha and backbone RMSDs were the same. For the clade A/E-NP-005114 complex, the RMSD of the protein backbone was observed to increase steadily till about 20 ns, where it stabilized with an average RMSD of 2.5 Å. At ~35 ns, the RMSD rose to 3.0 Å and remained stable with an average of 2.75 Å till the end of the 100 ns period. The ligand (NP-005114) was observed to remain stable throughout the 100 ns simulation period. For about 20 ns, the NP-005114 had an average RMSD of 2.2 Å and increased to an average RMSD of 3.0 Å till the end. For the clade A/E-NP-007382 complex, the RMSD values of the backbone ranged between 2.0 and 3.25 Å. The clade A/E-NP-007382 complex maintained an average RMSD of 3.0 Å throughout the 100 ns simulation period. NP-007382 had the least ligand RMSD, maintaining an average of 0.8 Å throughout the simulation. For the clade A/E-NP-007422 complex, the backbone’s RMSD rose to ~3.25 Å around 20 ns and then fell to 2.75 Å and remained stable till ~55 ns, where the RMSD fell to 2.5 Å, which was maintained till the end of the 100 ns simulation period. The RMSD of the ligand (NP-007422) was also observed to increase and then maintained stability till about 40 ns with an average ligand RMSD of 3 Å and then rose to 4.75 Å, maintaining this average till 100 ns. The clade A/E-NP-008297 complex demonstrated a very stable RMSD throughout the 100 ns simulation period with RMSD (average RMSD of 3 Å) ranging from 1.5 to 3.5 Å (Figure 5).

#### 2.1.10. RMSD of HXB2-Ligand Complexes

The protein RMSD was observed to stabilize after 2.5 ns with an average of 3.2 Å till about 12.5 ns, where fluctuations were observed. The protein RMSD stabilized again with an average value of 4.2 Å and remained consistent till the end of the 20 ns simulation period. For the HXB2-NP-008297 complex, stability was observed after 1.5 ns with an average RMSD of 5 Å, and this remained constant till about 7.5 ns. A reduction in RMSD was then observed with an average of 4 Å till after 12.5 ns, where fluctuations were observed. The protein achieved stability with an RMSD of 4.2 Å at 17.5ns till the end of the simulation. In the case of the HXB2-NP-007422 complex, the RMSD values ranged between 0.8 and 10.5 Å. The system exhibited fluctuations till about 7 ns and remained stable with an average RMSD of 6.1 Å till after 12.5 ns, where minimal fluctuations were observed. The system regained stability after 15 ns till the end of the simulation period with an average RMSD of 7.5 Å. Significant fluctuations were observed in the HXB2-NP-005114 complex throughout the simulation. However, the HXB2-NP-005114 complex tends to be stabilized at 15 ns till the 20 ns with an average RMSD value of 5.8 Å (Figure S8). 

#### 2.1.11. RMSF of Clade A/E-Ligand Complexes

To identify the movement of the protein residues in the active site and conformational changes, RMSF was calculated. All four clade A/E-ligand complexes had fluctuations in similar regions. Leu122 to Pro206 and Pro299 to Ile326 were observed to have shown high fluctuations in all four complexes. Clade A/E-NP-007382 and clade A/E-NP-008297 also demonstrated comparatively higher fluctuations around residues Gly397 to Gly412 and Gly459 to Ser464, respectively. The higher RMSF demonstrates that these residues are undergoing conformational changes during the simulation. Notably, however, the overall trend suggests greater flexibility of the residues but a stable binding of the compounds since they present uniform fluctuations in most of the residues (Figure S9). 

#### 2.1.12. RMSF of HXB2-Ligand Complexes

The RMSF plots revealed that all four compounds elicited some degree of fluctuations in similar regions of the HXB2 protein. Notably, there were fluctuations between residues Lys130 to Ala204 and Thr303 to Asn325 in all the HXB2-ligand complexes, with the latter exhibiting a greater degree of fluctuation. Similarly, the higher RMSF demonstrates that these residues are changing their conformation during the simulation. The overall trend observed here again suggests greater flexibility of the residues but a stable binding of the compounds since most of the interacting residues exhibited uniform fluctuations (Figure S10).

#### 2.1.13. Clade A/E-Ligand Complex Molecular Interaction

**Figure 6:**
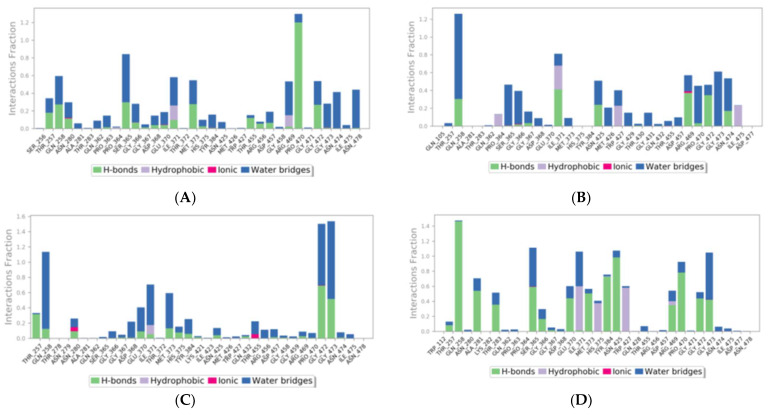
Analysis of the molecular interactions and the type of contacts with clade A/E throughout MD simulation. Normalized stacked bar chart of clade A/E residues interacting with (**A**) NP-005114, (**B**) NP-007382, (**C**) NP-007422, and (**D**) NP-008297. Hydrogen bonds, hydrophobic bond, ionic interactions, and water bridges are represented as green, grey, red, and blue, respectively.

For the clade A/E-NP-005114 complex, NP-005114 interacted with Thr258, Ser365, Ile371, Met373, Arg469, Pro470, Gly472, Asn474, and Asn478 for more than 40% of the simulation period. It was also observed to interact with residues Thr257, Gln258, Met373, Thr455, Arg456, Pro470, and Gly472, mainly via hydrogen bonds. Ile371 and Arg469 were also involved in hydrophobic contacts with NP-005114. Pro470 was observed to interact with NP-005114 with an interaction fraction more than 1.0 (Figure 6). For more than 40% of the 100 ns simulation period, NP-007382 formed interactions with Gln258, Ser365, Gly366, Ile371, Asn425, Trp427, Arg469, Pro470, Gly472, Gly473, and Asn474. Ile371, Asn425, Arg469, and Gly472 were mostly involved in hydrogen bonding. Ile371 was involved in both hydrogen and hydrophobic interactions. NP-007422 interacted with Gln258, Glu370, Ile371, Met373, Gly472, and Gly473 for more than 40% of the period. It was also observed to interact with Gln258, Gly472, and Gly473 with an interaction fraction greater than 1.0. NP-007422 was observed to interact with the clade A/E by forming hydrogen bonds with Thr257, Gln258, Asn280, Glu370, Ile371, Met373, His375, Tyr384, Asn425, Gln432, Asp457, Arg469, Gly472, and Gly473 during the simulation. For the clade A/E-NP-008297 complex, Gln258, Ala281, Thr283, Ser365, Glu370, Ile371, Thr372, Met373, His375, Tyr384, Asn425, Trp427, Arg469, Pro470, Gly472, and Gly473 formed contacts with NP-008297 for more than 40% of the 100 ns simulation period. Ile371, His375, Trp427, and Arg469 were involved in hydrophobic interactions while Thr257, Gln258, Ala281, Thr283, Ser365, Gly366, Glu370, Met373, Tyr384, Asn425, Arg469, Pro470, Gly472, and Gly373 were mainly involved in hydrogen bonds. The interaction analysis shows on average 60% of the ligands are involved in interactions and each molecule has a few unique interactions. This analysis strongly supports the scope of feature amalgamation as well as rational improvement to design more specific derivatives.

#### 2.1.14. HXB2-Ligand Complex Molecular Interaction

NP-005114 was observed to interact with Asn280, Trp427, Thr455, Arg456, and Asp457 mainly via hydrogen bonding during the simulation. NP-007382 formed bonds with Ala281, Lys282, Asn425, and Trp427 for more than 40% of the simulation time. It interacted with Ala281 and Lys282 mainly via hydrogen bonds and interacted mainly with Trp427 via hydrophobic bonds. NP-008297 also formed contacts with Thr283, Ser365, Gly366, Asp368, Ile371, Asn425, Pro470, and Asp477 for more than 40% of the 20 ns simulation period. It interacted with Thr283, Gly366, Asn425, and Asp477 mainly via hydrogen bonding. Moreover, it interacted mainly with Ile371 and Trp427 via hydrophobic contacts and Asp368 via an ionic bond. Asp368 had interaction fraction values of approximately 1.75, signifying that Asp368 interacted with HXB2 for more than 100% of the simulation period due to multiple simultaneous formations of interactions with NP-008297 [41]. NP-007422 was observed to form bonds with Ser365, Thr455, Arg456, Asp457, Gly458, and Asp474 for more than 40% of the 20 ns simulation period. NP-007422 interacted mainly with Asn280, Thr455, Arg456, Gly458, and Asp474 via hydrogen bonding. The formation of multiple hydrogen bonds between HXB2 and NP-007422 could influence its activity [42] (Figure S11). The interactions show clade independent interactions, and differences seen are due to differences in the conformations.

#### 2.1.15. Origin and Sources of Selected Compounds

Of the nine selected natural product-derived compounds, six were obtained from plants, three from micro-organisms, and one was a synthetic fragment of the natural compound (Table 4).

#### 2.1.16. Pharmacological Profiling 

The pharmacological and physicochemical properties of the nine selected compounds were obtained from SwissADME (Table 5 and Table 6). The compounds have molecular weights between 335.72 and 895.09 Dalton. Rotatable bonds are predictors of ligand conformational flexibility and small compound bioavailability [43], with rotatable bonds less than or equal to seven being ideal in drug discovery; eight of the compounds fell within this range. Pharmacological indices such as lipophilicity, solubility, gastrointestinal (GI) absorption, brain–blood barrier (BBB) permeant, P-glycoprotein (P-gp) substrate, and Cytochrome 450 inhibitor were used to predict the absorption, distribution, metabolism, and excretion (ADME) profiles of the compounds which describe their pharmacokinetic properties. The lipophilicity value for the selected compounds was between −0.98 and 1.91. The ideal lipophilicity value of compounds lies between 1 and 3 [44], with only four of the selected compounds falling within this range. Eight of the compounds were predicted to be soluble in water (Table 6). The summation of all the polar atoms of a molecule is defined by the topological polar surface area (TPSA) [45]. The TPSA value is a metric used to define cell permeability by a drug. The ideal TPSA usually falls within 140 to 90 Ǻ, above 140 Ǻ indicates poor permeability into a cell [46], and below 90 indicates penetration into the blood–brain barrier (BBB) [47]. None of the compounds fell within this range. Furthermore, none of the test compounds were BBB permeants and inhibitors of the CYP 450 enzyme (Table 6). However, nine of the compounds had low GI absorption and were substrates of the P-gp transporter (Table 6).

#### 2.1.17. Toxicity Profiling of the Selected Compounds

The result of the toxicity profile of the selected compounds predicted using ProTox-II is summarized in Table S2 [48]. ProTox-II generates the toxicity profiles of small compound molecules by measuring parameters such as immunotoxicity, hepatotoxicity, cytotoxicity, mutagenicity, carcinogenicity, and toxicity class [48]. Toxicity class ranges from 1–6 (1 = highly toxic/fatal, 6 = least toxic) with probability range of 0–1 (0 = not likely, and 1 = very likely). Eight of the compounds belonged to the less toxic drug class associated with high LD_50_, except for NP-007422, which belongs to toxicity class 2 (Table S2). Moreover, eight of the compounds were inactive for carcinogenicity and cytotoxicity, whilst seven were predicted as immunotoxins. None of the compounds was hepatotoxic or mutagenic.

### 2.2. Cell-Based Viral Infectivity Inhibition Assay

#### 2.2.1. In Vitro Cell Cytotoxicity Assessment

To evaluate the cytotoxic effect of the test compounds on target TZM-bl cells, cell viability was determined using the Alamar blue assay. Compounds FGR-0075, NP-00603, NP-00088, NP-001800, NP-005114, NP-008297, and NP-007422 had no cytotoxic effect on TZM-bl cells at the concentration tested (Figure S12). Compounds NP-007382 and NP-004255 exhibited cytotoxic effects. The CC_50_ was calculated in Figure S13. Ursolic acid, a known cytotoxic compound, was used as a positive control.

#### 2.2.2. Determination of 50% Cytotoxicity Concentration (CC_50_)

To determine the 50% cytotoxic concentration (CC_50_) of compounds that had a cytotoxic effect on the TZM-bl cells, a non-linear dose–response regression analysis was used to calculate CC_50_. CC_50_ was defined as the concentration of a compound that is required to reduce viable cells by 50%. Compound NP-007382 had CC_50_ of 47.6 µM (28.4 µg/mL), while NP-004255 had CC_50_ of 14.4 µM (9.1 µg/mL), respectively (Figure S13).

#### 2.2.3. Pseudotype Virus (PV) Titration and Determination of 50% Tissue Culture Infectivity Dose (TCID_50_)

PV titration was carried out to determine the number of viable PVs produced. A viable PV was defined as a PV that can infect a target cell and integrate the luciferase reporter gene, leading to the expression of the reporter gene. The quantity of light produced by the luciferase enzyme (relative light unit RLU) is measured with a luminometer and directly correlates with the number of viable PVs in a harvest. TCID_50_ was defined as the number of PVs that can infect 50% of target cells (TZM-bl cells). A positive infection was defined as one with 2.5 times the average RLU of the “cells only” control wells. TCID_50_ was calculated in the TCID_50_ MACRO sheet, using the modified Spearman and Karber methods [49], and was determined to be 5657 TCID_50_/mL. The cut-off for the luminescence was set at 10,000 RLU, and the recommended volume of PVs that will produce 10,000 RLU was calculated to be 105 µL.

#### 2.2.4. Viral Infectivity Inhibition Assay

**Figure 7:**
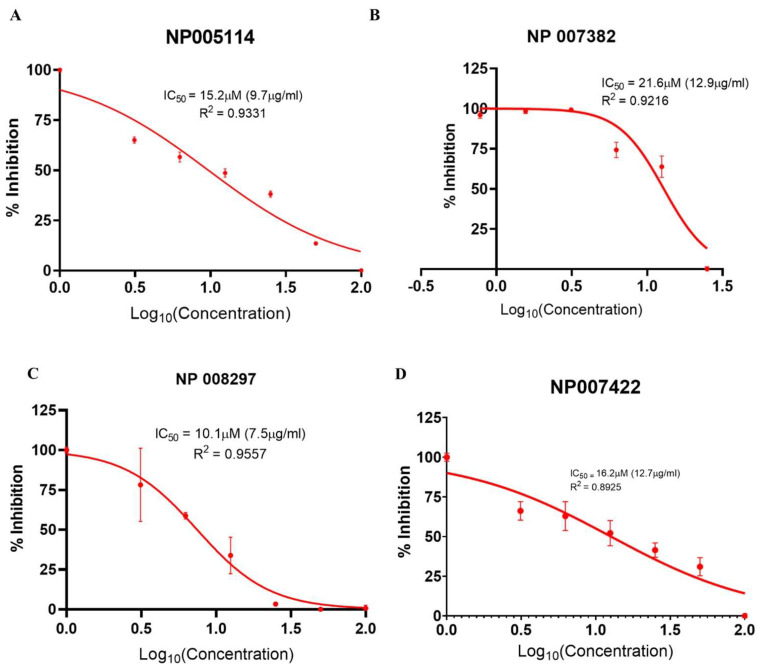
Cell-based viral infectivity inhibition assay. Nonlinear regression plot of percentage inhibition (response) against the Log_10_ of compound concentration (dose) at a 95% confidence interval. (**A**) NP-005114 had IC_50_ of 15.2 µM (9.7 µg/mL), (**B**) NP-007382 had IC_50_ of 21.6 µM (12.9 µg/mL), (**C**) NP-008297 had IC_50_ of 10.1 µM (7.5 µg/mL), and (**D**) NP-007422 had IC_50_ of 16.2 µM (12.7 µg/mL). Error bars represent the standard error of the mean (SEM) of triplicate wells.

The concentration of a compound where the RLU (response) is reduced by 50% relative to the “cells + PVs” control was defined as the 50% inhibition concentration (IC_50_) of that compound. Only four (NP-005114, NP-008297, NP-007422, and NP-007382) out of the nine tested compounds exhibited a dose-dependent inhibition of viral infection of TZM-bl cells. NP-005114 had IC_50_ of 15.2 µM (9.7 µg/mL, R^2^ = 0.9331, [95%CI = 7.2–12.9]), NP-007382 had IC_50_ of 21.6 µM (12.9 µg/mL, R^2^ = 0.9216, [95%CI= 10.2–15.9]), NP-008297 had IC_50_ of 10.1 µM (7.5 µg/mL, R^2^ = 0.9557, [95%CI = 6.0–9.2]), and NP-007422 had IC_50_ of 16.2 µM (12.7 µg/mL, R^2^ = 0.8925 [95%CI = 8.7–18.3]) (Figure 7A–D). Compound NP-004255, however, exhibited a dose-independent inhibition of viral entry (Figure S14). There was no observed viral inhibition by compounds FRG-00075, NP-005003, NP-000088, and NP-001800 (Figure S14).

#### 2.2.5. Selectivity Index

The selectivity index (SI) parameter is used to access a compound’s in vitro efficacy in the inhibition assay by evaluating the cytotoxic effect versus the antiviral effect. SI was defined as the ratio of the 50% cytotoxic concentration (CC_50_) to the 50% inhibitory concentration (IC_50_) and was calculated for compound NP-007382 with CC_50_ of 47.6 µM and IC_50_ of 21.6 µM. The SI was determined as 2.2.

## 3. Discussion

This study used a pharmacoinformatics approach to identify compounds derived from natural products (plants and micro-organisms) that mimic VRC01 bNAbs by interacting with similar binding sites as VRC01 (CD4-binding site) on the HIV gp120. The CD4-binding site (CD4-bs) is a conserved region on the HIV-1 gp120 that is crucial for the CD4-gp120 interaction required for successful HIV-1 infection. Out of 27, 824 compounds analyzed, nine compounds (NP-008297, NP-004255, NP-000088, NP-007422, NP-005114, NP-007382, NP-005003, NP-001800, and FRG-00075) that interacted with the CD4-bs of gp120 in silico were confirmed in vitro using pseudotype virus technology. While the compounds’ purity range was around 70%, natural compounds are secondary metabolites that are formed by a variety of cascades. They have similar properties with different compounds upstream of the synthetic pathway as well as other metabolic branches of similar compounds derived from similar precursors. This makes isolating a single compound very difficult. The best example is curcumin; while there is a known structure for curcumin, the preparations, even pharmaceutical ones, are a mixture of many curcuminoids (77% curcumin, 17% desmethoxycurcumin, and 3% bisdemethoxycurcumin) with slight variations in structure but highly similar chemical properties, making pure curcumin isolation a high-loss process [50]. Similarly, we expect these compound mixtures to have highly similar compounds, and many of them might be sharing the pharmacophore; thus, what we are seeing could be a cumulative effect.

In silico analysis of the test compounds revealed that the test compounds had high binding affinities to the critical amino acid residues of CD4-bs of clade A/E recombinant and clade B HIV-1 gp120. The ligands were found to be firmly docked in the binding domain of CD4-bs of both recombinant clades A/E and clade B (HXB2) HIV-1 gp120 (Figure 3A, Figure 4A and Figure S3). The binding energies of the screened ligands ranged from −8.2 to −4.8 kcal/mol for the gp120 of clade B and −10.3 to −7.4 kcal/mol for recombinant clade A/E gp120 (Table 2). Analysis of the molecular interaction between the test compounds and HIV-1 gp120 showed that the compounds exhibited intermolecular hydrogen and hydrophobic bonding with key amino acid residues of the CD4-bs gp120 of both clade B (Figure 4B and Figure S7) and recombinant clade A/E (Figure 3B and Figure S4). HIV-1 clade A/E and clade B gp120 were used as the protein receptor for the virtual screening to compare the protein–ligand interaction of our test compounds to the gp120-VRC01 [17] and CD4-gp120 [51] interactions, respectively. Protein–ligand interaction analysis showed that NP-005114, NP-008297, NP-007382, and NP007422 partially mimic CD4 and VRC01 in their interaction with the CD4-binding sites of HIV-1 gp120. The stability of the protein–ligand complexes was assessed by subjecting the complexes to molecular dynamics simulations. For all the complexes, the RMSDs of the protein backbone, side chains, and heavy atoms demonstrated similar trends. The RMSD trajectory plots of the clade A/E complexes indicated stability in all protein–ligand complexes by the end of the 100 ns simulation. The RMSD plots also revealed that the HXB2-NP-007382, HXB2-NP-008297, and HXB2-NP-007422 complexes demonstrated more stability than the HXB2-NP-005114 complex. Overall, these results highlight the binding stability of the complexes and underscore the mechanistic basis for the inhibition of HIV-1 entry. A previous study showed that the conformational equilibrium for the HXB2 backbone is reached after 5 ns, similar to the results obtained herein [52]. 

The lead compounds had intermolecular hydrogen and hydrophobic interactions with the critical amino acid residues of the CD4-bs, which are important for the binding of VRC01 and CD4 to gp120. Compounds NP-005114, NP-008297, NP-007382, and NP007422 had biomolecular hydrogen and hydrophobic interactions with Asp368 gp120, Glu370 gp120, Ile371 gp120, Asn425 gp120, Met426 gp120, Trp427 gp120, Gly473 gp120, Met475 gp120, and Asp457 gp120, which are critical and conserved residues in the VRC01-gp120 interaction [17] and Phe43 cavity. The Phe43 cavity is a functionally crucial and conserved pocket on HIV gp120, where CD4 binds [51,53,54] and alterations in the amino acid residues lead to a decrease in CD4 binding [55]. The molecular interaction profiles of the protein–ligand complexes, which were assessed using MD simulation to determine the nature of the bonds formed between the protein and the ligand in a dynamic environment, are consistent with what were observed during molecular docking. This is indicative of the predicted stability of the intermolecular hydrogen and hydrophobic bonds formed between the protein and ligand, which partially mimic the CD4–gp120 and VRC01-gp120 interactions. Many small compounds that have been developed to target the CD4-bs of HIV-1 gp120 [56] interact with the Phe43 cavity in CD4-bs and have demonstrated broad activity against various strains of HIV-1 entry into target cells [57]. The interactions of these compounds with gp120 of clades A/E and B indicate possible conservation of the CD4-bs [58], despite constant variation of the HIV envelope protein due to mutations caused by error-prone HIV reverse transcriptase. Although it is also indicative of the possibility of interactions with gp120 from several strains of HIV, more work needs to be carried out to determine the interaction of the lead compounds with other HIV clades. 

Small compounds with the desired bioactivity are assessed for drug-likeness (Lipinski’s rule of five) using their physicochemical properties. Lipinski’s rule allows not more than 5 and 10 hydrogen bond donors and acceptors, respectively, LogP ≤ 5, and molecular mass (MW) of ≤500 Daltons [59]. Compounds NP-005114, NP-00892, and NP-007422 violated three of Lipinski’s rules (MW > 500, H-bond donors, and acceptors not more than 5 and 10, respectively), while compound NP-007382 violated two rules (MW>500, H-bond acceptors >10) (Table 5). The implication of violating Lipinski’s rules is that the compounds are predicted to have poor pharmacokinetic properties (ADME) and oral bioavailability. However, Lipinski’s rule does not predict pharmacological activity [59]. 

The absorption, distribution, metabolism, and excretion (ADME) properties of the compounds describe their pharmacokinetics. Parameters including lipophilicity, solubility, gastrointestinal (GI) absorption, brain–blood barrier (BBB) permeant, P-glycoprotein (P-gp) substrate, and Cytochrome 450 inhibitor are also used to predict pharmacokinetic properties (Table 6). Lipophilicity is an indication of the solubility, permeability, selectivity, and promiscuity of a drug compound. Lipophilicity > 5 has been associated with poor receptor selectivity, high metabolic turnover, low solubility, and poor absorption. However, compounds with low lipophilicity values are also linked to poor ADME properties [44]. The ideal lipophilicity value lies between 1 and 3; only NP-008297 and NP-005114 fell within this range. All lead compounds have low GI absorption, possibly due to the complex interplay of many factors such as molecular weight, cell permeability, and solubility of the compounds; however, these properties can be optimized in the developmental stage of drug discovery [60,61]. TPSA value is a metric used to define cell permeability by a drug compound. The ideal TPSA usually falls within 140 to 90 Ǻ, above 140 Ǻ indicates poor permeability into a cell [44], and below 90 indicates penetration into the blood–brain barrier (BBB) [47]. None of the compounds fell within this range. The blood–brain barrier (BBB) permeability index indicated the ability of the drug compound to be deposited into the central nervous system (CNS) [62]; none of the compounds was BBB permeant (Table 6). P-glycoprotein (P-gp) is a transmembrane efflux pump that is important in the efflux and uptake of the drug compound [63]. NP-008297 and NP-007382 were substrates of the P-gp transporter. Cytochrome P450 (CYP450) enzymes play a significant role in drug metabolism, excretion, and drug–drug interactions [64]. Compounds that inhibit CYP enzymes interfere with the metabolism of other drugs that are activated by the enzyme. None of the compounds were inhibitors of the CYP 450 enzymes. There is scope for rational improvement of the leads to further derive an inhibitor with a better bioavailability profile.

The toxicity profile of the selected compounds predicted using ProTox-II is summarized in Table S2 [48]. ProTox-II generates the toxicity profiles of small compound molecules by measuring parameters such as immunotoxicity, hepatotoxicity, cytotoxicity, mutagenicity, carcinogenicity, and toxicity class [48]. Toxicity class ranges from 1 to 6 (1 = highly toxic/fatal, 6 = least toxic) with probability range of 0–1 (0 = not likely, 1 = very likely). All the compounds except NP-007422 and NP-005003 belonged to the less toxic drug class associated with LD_50_ ≥ 1000 (Table S2). None of the compounds were shown to exhibit hepatotoxicity and mutagenicity. FRG-00075 was predicted as carcinogenic whilst NP-005114 and FRG-00075 were predicted to be immunotoxins. NP-007422 was also predicted to be cytotoxic and highly lethal. Further research is required to optimize the structures of the potential leads using the pharmacophores to circumvent these toxicity predictions. Worthy of note is the disparity between the in silico predicted toxicity profile and the observed in vitro cytotoxicity. Despite compounds NP-004255 and NP-007382 being predicted as non-cytotoxic in HeLa cell lines, they demonstrated some cytotoxicity in vitro. This could be because ProTox-II predicts by using molecular and pharmacophore similarity of lead compounds to known compounds in its database, hence a given limitation if the lead compound does not have high similarity to known toxic compounds in the database [65]. Further transcription profiling may reveal the mechanism/off-target behind observed cytotoxicity. 

Although the in silico predictions are beneficial in identifying possible bioactive compounds, there is a need for further confirmation using experimental methods. Inhibition of viral entry by the test compounds was examined using molecularly cloned HXB2 PV. Only four compounds, NP-005114, NP-007382, NP-008297, and NP-007422, exhibited a dose-dependent inhibition of clade B (HXB2) env PV entry into TZM-bl cells, with IC_50_ values of 15.2 µM (9.7 µg/mL), 21.6 µM (12.9 µg/mL), 10.1 µM (7.5 µg/mL), and 16.2 µM (12.7 µg/mL), respectively. Compound NP-004255, however, exhibited a dose-independent inhibition of viral entry (Figure S14). The antiviral compound NP-004255 could not be differentiated from the cytotoxic effect on the target cells. There was no observed viral inhibition by compounds FRG-00075, NP-005003, NP-000088, and NP-001800 (Figure S14). 

From previous literature [20,66,67,68], the IC_50_ values of VRC01 in the neutralizing panel of clade B HIV and clade B env PV ranged from 0.1 to 50 µg/mL. The IC_50_ of NP-005114, NP-008297, and NP-007382 obtained from this study is well within this range. Several small compound HIV-1 entry inhibitors, targeting the CD4-bs of the HIV-1 gp120 [56], have been discovered recently. However, only BMS-663068 and NBD-556, as well as their derivatives, have shown good antiretroviral activity and are in the preclinical and clinical trials of the drug development process [69,70,71]. BMS-663068 is a more potent derivative of BMS-626529, with inhibition to viral entry against a panel of PVs at IC_50_ of between 0.0001 and 9.5 µg/mL with varying sensitivity towards different clades of HIV-1 [72]. NBD-556 and its derivatives were shown to inhibit entry of HIV-1HXB2 PVs at IC_50_ of 20.0 µM [73]. Further testing is needed with complete viruses and different clades, especially newly emerged clade C [74].

Compound NP-005114 was extracted from the seed of *Terminalia chebula* (Table 4). *T. chebula* is a medicinal plant popularly referred to as the “king of medicine” due to a wide array of bioactivity of compounds extracted from the plant. Compounds extracted from *T. chebula* fruit are HIV-1 integrase and reverse transcriptase inhibitors [75,76]. So far, no compound extracted from *T. chebula* has been characterized as an HIV-1 entry inhibitor [77,78]. NP-005114 has exhibited an HIV-1 entry inhibitory property. NP-008297 was extracted from the leaf of *Ginkgo biloba*. *G. biloba* crude extracts have shown activity against HIV-1 reverse transcriptase RNase H and protease [77,79]. This study has shown the HIV-1 entry inhibition activity of NP-008297. Compound NP-007422 was derived from *Withania somnifera*, popularly known as Ashwagandha. Ashwagandha herb is popular for its anxiolytic and adaptogenic effects [80] and has been found to reverse neuron cells toxicity induced by β-Amyloid in HIV-associated neurocognitive disorders (HAND) [81]. Unlike the others, NP-007382 was extracted from actinomycetes, which are micro-organisms that have been a source of active compounds against HIV-1 [77].

## 4. Materials and Methods

### 4.1. In Silico High-Throughput Screening of Natural Compound Mimetics of Vrc01

#### 4.1.1. Protein Structure Extraction

The 3D structure of crystallized antigen-binding fragment (Fab) of the broadly neutralizing antibody, VRC01, in complex with core gp120 of HIV-1 clade A/E recombinant 93TH057 (PDB ID: 3NGB), with a resolution of 2.68 Å was downloaded from the Protein Data Bank (PDB). The core gp120 trimer consisting of outer and inner domains with truncated N- and C-termini, as well as V1/V2 and V3 variable loops, were extracted from the complex as protein chains A, G, and D using PyMOL 1.74 (Schrödinger, Inc., NY, USA) [82].

#### 4.1.2. Determination of gp120 Core Residues of Interaction with VRC01

VRC01 is a CD4-binding site (CD4bs) broadly neutralizing antibody (bNAb) which makes contact with 98% of gp120 sites of vulnerability [17]. The gp120 vulnerability site is the highly conserved CD4 receptor contact site on gp120 outer domain. The gp120 amino acid residues that interact with VRC01 and CD4 were obtained elsewhere [17].

#### 4.1.3. Receptor Molecular Dynamics (MD) Simulation

MD simulation of the HIV-1 gp120 core (PDB ID: 3NGB) was carried out to determine the conformation, stability, and dynamics of the structure. A 10 ns MD simulation was performed using GROMACS 5.1.4 [83]. The CHARMM27 force field was adopted to prepare the topology input file for the protein. The protein was solvated by SPCE water molecules and immersed in a 1 nm thick cubic periodic water box. Before the simulation, a short minimization of 500 steps using the steepest descent method was carried out to remove possible distortion in the protein structure caused by the addition of water to the system. Eight Cl^-^ ions were added to neutralize the system. The system was equilibrated at a temperature of 300 K and normal pressure for 50 ps to restrain the heavy atoms of the proteins to their starting position to allow water molecules to saturate the system. The final production simulation was performed for 10 ns under similar conditions as the equilibration steps. The root-mean-square displacement (RMSD) of the minimized protein heavy-atom concerning the resolved X-ray structure was calculated and plotted using GRACE 5.1.4 [84]. The final minimized GROMACS protein file was visualized with Visual Molecular Dynamics (VMD) 1.9.3 version [85] and saved as frames in PDB format for further analysis.

#### 4.1.4. Receptor Preparation

The minimized HIV-1 gp120 was prepared for molecular docking using AutoDockTools version 4.2.6 (Scripps Research, La Jolla, CA, USA). Water molecules were removed from the structure, polar hydrogen atoms were added, and non-polar hydrogens were merged with a parent carbon atom. Gasteiger partial charges of the atoms were calculated and added. The protein file was then converted to AutoDock compatible format (pdbqt). The energy grid box was set around the conserved residues of HIV-1 gp120 with a dimension of 42 Ǻ × 50 Ǻ × 56 Ǻ, and coordinates of X = 52.318 Ǻ, Y = 26.081 Ǻ, and Z = 46.493 Ǻ for the receptor macromolecules.

#### 4.1.5. Ligand Compound Library Generation

Libraries of natural compounds were obtained from three commercially available natural product-derived compound databases consisting of AnalytiCon Discovery, Specs, and InterBioScreen (IBS). Structures of compounds retrieved from these databases were downloaded as a single structure-data file (SDF) format. 

#### 4.1.6. Ligands Preparation 

SDF format files of the ligand libraries were split into individual compounds using a split utility module in Open Babel 2.3.1 [86] accessible via the PyRx 0.8 [87]. The compounds were optimized and converted to AutoDock compatible (PDBQT) format. A total of 27,824 ligands were minimized for molecular docking.

#### 4.1.7. High-Throughput Virtual Screening 

High-throughput virtual screening of the compound libraries was performed on a Linux Operating System High-Performance Computing System (Zuputo) hosted by the West African Centre for Cell Biology of Infectious Pathogens (WACCBIP). AutoDock Vina 1.1.1 (Scripps Research, La Jolla, CA, USA), a molecular docking tool, was used for the virtual screening of compound libraries. AutoDock Vina utilizes rigid receptor and flexible ligand docking mode and empirical scoring function to rank protein–ligand complexes [88]. The previously prepared HIV-1 gp120 protein’s PDBQT file with defined grid box dimensions (42 Ǻ × 50 Ǻ × 56 Ǻ, coordinates of: x = 52.318 Ǻ, y = 26.081 Ǻ, z = 46.493 Ǻ) and exhaustiveness set value 9 (amount of computational effort used during a docking experiment; default value 8), along with all optimized ligands, was used for docking. A custom bash script was prepared for the docking procedures. All virtual screening results were aggregated and written to a single log file. The log file contained binding energies for each of the docked complexes.

#### 4.1.8. Virtual Screening Result Analysis

AutoDock Vina scores the binding affinities of the ligand to the receptor using empirical data obtained from the summation of energies contributed by the receptor and ligand interactions, which is calculated as the total atom pair distance-dependent interactions of the protein and ligand [89]. The binding energy values are inversely related to the binding affinity, i.e., the lower the binding energy value, the higher the binding affinity. The single log file containing the binding energies in (kcal/mol) of all processed ligands was extracted into a comma-separated value (CSV) file format and was analyzed using Microsoft Excel 2016 (Microsoft Corporation, One Microsoft Way, Redmond, WA, USA). The docked poses of each ligand were visualized using PyMOL. The poses with the best fit in the binding site with the lowest binding energies were selected for further analysis.

#### 4.1.9. Protein–Ligand Interaction Profiling

The protein–ligand complex interaction profiles were determined using LIGPLOT version 1.4.5 [39]. LIGPLOT analyzes the molecular interaction between proteins and ligands and generates 2D schematic representations of the interactions in terms of bond types. The bond types include hydrogen and hydrophobic interactions, as well as bond lengths and interacting residues. Hydrogen bond interactions are represented by broken green lines of distinct length, while arcs with spikes pointing towards the ligands represent the hydrophobic interactions. 

#### 4.1.10. HIV-1 HXB2 Envelope Protein

Human immunodeficiency virus clade B clone 2 (HIV HXB2) was used as the reference HIV strain. HXB2 envelope protein was used in the production of HIV-1 PVs utilized in the cell-based viral infectivity inhibition assay. Virus-like particles are not full-length genomes and lack nonstructural proteins required for viral replication and jumping elements for recombination and thus are categorized in RG1 of biosafety handling.

#### 4.1.11. Determination of HIV-1 HXB2 Envelope Protein Structure

The nucleic acid sequence of HXB2 envelope protein (gp160) that was cloned into the pCAGGGS plasmid was retrieved from the HIV sequence database. The nucleic acid sequence was converted to an amino acid sequence using the ExPASy Translate tool, which translates nucleic acid sequences into an amino acid sequence. The HXB2 gp160 amino acid sequence was retrieved from the ExPASy Translate tool server as a FASTA file and used as a query for BLASTP via the ExPASy platform. BLASTP is used to query the protein sequence database to identify similar sequences. The protein sequence with the highest similarity score to our query protein sequence was retrieved from UniProt Knowledgebase (UniProtKB). UniProtKB is a comprehensive protein database that contains annotations and functional information of the proteins. A structure similarity search was performed using the UniProt ID of the protein sequence to retrieve the 3D protein structure (PDB ID: 3J70) from Protein Data Bank.

#### 4.1.12. Protein Structure Extraction and Energy Minimization of HXB2 gp120

The 3D structure of HXB2 gp160 with the entire variable regions in complex with CD4 and antibody 17b was retrieved from Protein Data Bank (PDB ID: 3J70) [90]. The gp120 trimer was extracted from the protein complex using PyMOL 1.74 [82] as protein chains D, P, and U. The energy of the extracted gp120 protein was then minimized using Swiss-Pdb Viewer 4.2 [91] with default settings. Swiss-PDB Viewer [91] utilizes the Groningen Molecular Simulation computer program package (GROMOS 43B1) [83] force field to minimize the energy of the protein and repair distorted geometries of the protein structure. The minimized protein was extracted and saved as a PDB file.

#### 4.1.13. Receptor Preparation

In preparation for molecular docking, the minimized HIV-1 HXB2 gp120 was optimized using AutoDockTools version 4.2.6 (Scripps Research, La Jolla, CA, USA). Water molecules were removed from the structure to prevent interference with the docking process. Polar hydrogens were added, and non-polar hydrogens were merged with a parent carbon atom. Gasteiger partial charges of the atoms were calculated and added. The protein file was then converted to AutoDock compatible format (pdbqt). The energy grid box was set around the conserved residues of HIV-1 gp120 at dimensions (54 Ǻ × 40 Ǻ × 44 Ǻ) with coordinates of X = 249.640 Ǻ, Y = 230.870 Ǻ, and Z = 198.249 Ǻ. After optimization, the protein was used as a receptor for molecular docking. The resultant complex was analyzed, and the protein–ligand interaction was profiled using LIGPLOT [39].

#### 4.1.14. The Root-Mean-Square Deviation (RMSD) of the Complexes

The stability of the protein–ligand complexes was assessed using molecular dynamics simulations performed with the Desmond module in Schrödinger Suite (Schrödinger Release 2021-2, Schrödinger, LLC, New York, NY, USA). A 20 ns MD simulation was performed for the HXB2-ligand complexes since previous study has shown that the backbone reaches conformational equilibrium after 5 ns [52]. However, for clade A/E-ligand complexes, extended 100 ns MD simulations were performed. The MD simulations were aimed at understanding the stability, dynamic fluctuations, and molecular interactions in the protein–ligand complexes. The docked conformers of NP-005114, NP-007382, NP-007422, and NP-008297 with each of the receptors were used for the MD simulations.

#### 4.1.15. The Root-Mean-Square Fluctuation (RMSF) of the Complexes

The RMSF trajectories of the complexes were also investigated. The RMSF plot provides information on the flexibility of the various regions of a protein, which is related to the crystallographic B-factors [92]. Protein regions with significantly high fluctuations represent the areas involved in ligand binding and catalysis [93].

#### 4.1.16. Molecular Interactions under Dynamic Simulation

Molecular interactions and the type of bonds were investigated to understand the contacts between the proteins and stability of the complex in presence of water (TIP3p) and ions similar to physiological condition (0.15 M NaCl) and each ligand throughout the simulation period. Various types of interactions including hydrogen bonds, hydrophobic bonds, ionic bonds, and water bridges were formed during simulation, which are represented in the stacked bar charts as green, grey, red, and blue, respectively. The stacked bar charts represent the bonds over the simulation trajectory. All the ligands kept the water from entering the binding site, indicating stable binding. Few water molecules participated in the complex, indicating more stability in physiological conditions. Moreover, the complexes transitioned to lower energy states without ligands flying off, validating the interaction and establishing ligand strain is far less than interaction energies. 

#### 4.1.17. Physicochemical, Pharmacokinetics, and Drug-Likeness Properties Prediction 

SwissADME (Swiss Institute of Bioinformatics, Amphipôle, Quartier UNIL-Sorge, 1015 Lausanne, Switzerland) was used to predict the relevant physicochemical, pharmacokinetics, and drug-likeness properties of the shortlisted ligands [94]. The Simplified Molecular Input Line Entry Specification (SMILES) formats of the query ligand files were used to generate parameters such as physicochemical properties, lipophilicity (trans-membrane movement), absorption, distribution, metabolism, and excretion (ADME) profiles. The others include gastrointestinal absorption, blood–brain barrier permeability, P-glycoprotein substrate, cytochrome enzyme inhibition, water solubility, and drug-likeness (Lipinski’s rule of 5).

#### 4.1.18. Toxicity Profile Prediction

The toxicity profiles of selected compounds were predicted using ProTox-II [48]. ProTox-II utilizes compound pharmacophore fingerprint, structural similarity, and machine learning models designed from both in vitro and in vivo assay data to predict immunotoxicity, hepatotoxicity, cytotoxicity, mutagenicity, and carcinogenicity. The toxicity profiles of the compounds have a probability range of 0–1 (0 = not likely, 1 = very likely), predicted lethal dose (LD_50_), and toxicity class which ranges from 1 to 6 (1 = highly toxic/fatal, 6 = least toxic).

### 4.2. Cell-Based Viral Infectivity Inhibition Assay

#### 4.2.1. Reagents

Complete cell culture medium was prepared using Dulbecco’s Modified Eagle Medium (DMEM) supplemented with L-glutamine, streptomycin, penicillin, and heat-inactivated fetal bovine serum (FBS), all from Thermo Fisher (Thermo Fisher, Waltham, MA, USA). 10X Trypsin-EDTA (0.5%), Phosphate Buffered Saline (PBS) and Dimethyl sulfoxide (DMSO) were purchased from Sigma Aldrich. 

#### 4.2.2. Plasmids

Plasmids p8.91, pCSFLW, and pCAGGS-HXB2 were donated by Dr. Edward Wright (University of Sussex, Brighton BN1 9QG, UK). The p8.91 plasmid described in PNAS by Naldidni et al., 1996 [95], expresses HIV gag-pol, tat, and rev. pCSFLW is a lentiviral vector expressing firefly luciferase, and pCAGGS-HXB2-env is a mammalian expression vector expressing HIV-1 envelope protein (gp160) clade B clone 2.

#### 4.2.3. Cell Lines

TZM-bl cells are HeLa cells with HIV-1-tat-directed luciferase reporter gene, engineered to express CD4+ receptors and co-receptors. HEK 293T/17 is an adherent human kidney cell line that is highly transfectable and capable of producing retroviruses in high titer. Both TZM-bl and HEK 293T/17 cell lines were donated by Dr. Edward Wright (University of Sussex, Brighton BN1 9QG, UK).

#### 4.2.4. Cell Culture

TZM-bl and HEK 293T/17 cells were cultured in Dulbecco’s Modified Eagle’s Medium (DMEM) containing 10% fetal calf serum + 50 unit/mL penicillin + 50 μg/mL streptomycin in a 10 cm culture dish containing 10 mL of media and incubated at 37 °C in 5% CO_2_. The cell monolayer was washed twice with PBS and treated with 1.5 ml of 1X trypsin-ethylenediaminetetraacetic acid (EDTA) to dissociate adherent cells. The re-suspended cells were seeded into 10 mL of new culture plates for transfection assay.

#### 4.2.5. Production of HIV Pseudotype Viruses (PV)

Clade B (HXB2) HIV-1 PV was used to test the ability of the compounds to inhibit HIV-1 entry into target cells. HIV PVs were produced by transfecting HEK293T-17 cells, using the protocol described by Longo and colleagues [96]. Briefly, plasmid DNA mix (pCAGGSHXB2, p8.91, and pCSFLW) and transfection reagent polyethyleneimine (PEI) mix from Polysciences, Inc. (Polysciences Inc., Warrington, PA, USA), were prepared in separate Eppendorf tubes containing serum-free media (OptiMEM). The contents of the two tubes were mixed and incubated for about 20 min and afterward added to HEK293T-17 cells. The transfection plate was incubated overnight at 37 °C (5% CO_2_). The media were changed after 14 h, with culture supernatant containing HIV-1 PVs harvested at 48- and 72-h post-transfection. 

#### 4.2.6. Determination of Tissue Culture Infectivity Dose (TCID_50_) of Harvested Pseudotype Virus (PV)

In a 96-well plate, 25 µL of harvested PVs was serially diluted (1/5 dilution) in quadruple across the plate (in complete media) and incubated at 37 °C (5% CO_2_) for 30 min. Approximately 2 × 10^4^ (100 µL) of TZM-bl cells was added, and the plate was incubated for 48 h at 37 °C (5% CO_2_). The control wells contain only TZM-bl cells. After 48 h, spent media were discarded, and 50 μL of a 50:50 mix of media and Bright Glo substrate was added to the well and incubated for 3 min. The luminescence of each well was quantified using the Promega GloMax® Explorer Multimode Microplate Reader (Promega Corporation, Madison, WI, USA) at a 0.3 shake rate. TCID_50_ was calculated in the TCID_50_ MACRO sheet using the modified Spearman and Karber method [49]. TCID_50_ was defined as the number of PVs that can infect 50% of target cells (TZM-bl cells). The cut-off for the luminescence was set at 10,000 RLU.

#### 4.2.7. Preparation of Test Compounds

The nine test compounds were purchased from the natural compounds screening libraries of AnalytiCon Discovery (https://ac-discovery.com/screening-libraries/ (accessed 18 February 2019), with purity ranging from 70 to 90 (Table 4). The compounds were prepared and diluted to 1 mg/mL in 10% DMSO. The final concentration of DMSO in the downstream analysis was 0.1% (v/v). The maximum concentrations of test compounds used for downstream assay have been summarized in Table S1.

#### 4.2.8. Evaluation of Compounds Cytotoxicity

Alamar blue assay was conducted to evaluate the effect of the test compounds on TZM-bl cells. A volume of 100 µL (approximately 2 × 10^4^ cells) of TZM-bl cells was seeded overnight in a 96-well plate, treated with serially diluted test compounds, and incubated for 48 h at 37 °C and 5% CO_2_. Positive controls were cells treated with ursolic acid, a known cytotoxic compound, negative controls were cells with no compound treatment, with compounds and media only serving as color control, and media only as blank. Absorbance was read at 570 nm after 48 h. The assay was set up in triplicates, and mean values were calculated for analysis. Cell viability was calculated relative to the absorbance of the negative control wells (TZM-bl cells only), which were set as 100% of absorbance, using the equation below

(1) $$\mathrm{Cell}\;\mathrm{viability}\;\left(\%\right)=\left[\frac{{\mathrm{Abs}}_{\mathrm{sample}\;–{\;\mathrm{Abs}}_{\mathrm{blank}}}}{{\mathrm{Abs}}_{\mathrm{Neg}\_\mathrm{control}\;–}{\;\mathrm{Abs}}_{\mathrm{blank}}}\right]\times 100\%$$

#### 4.2.9. Viral Infectivity Inhibition Assay

The ability of test compounds to inhibit HIV entry into target TZM-bl cells was tested in a viral infectivity inhibition assay previously described by [97]. Briefly, test compounds were serially diluted and incubated with HIV-1 PVs for 1 h at 37 °C, after which TZM-bl cells were added and incubated for 48 h at 37 °C. The controls included TZM-bl cells only (assay control), HIV PVs only (negative control), and HIV PVs with TZM-bl cells (positive control). The relative light unit (RLU) from the assay was read using the Promega GloMax® Explorer Multimode Microplate Reader (Promega Corporation, Madison, WI, USA). The IC_50_ was calculated using non-linear regression dose–response analysis with GraphPad Prism version 8 as described by [97], with slight modification. The modification was by normalization of the Y-axis (response) to 0% = PVs only control and 100% = cells + PVs only. A cells-only control was set up as background RLU.

## 5. Conclusions

To our knowledge, this is the first study on natural product-derived compounds that mimic HIV-1 broadly neutralizing antibody VRC01 by interacting with the CD4-bs of HIV-1 gp120 of recombinant clades A/E and B. The study combined varieties of in silico techniques including molecular docking and in vitro screening. We have identified four compounds with the potential to inhibit HIV-1 clade B (HXB2) entry into target TZM-bl cells. The study further identified novel insights into the binding mechanisms including potential residues critical for interactions using molecular dynamics simulations. The molecules were shown to have reasonably good pharmacological profiles and were predicted as drug-like. The molecules could serve as templates for the design of next-generation HIV-1 inhibitors of therapeutic importance. The study warrants the experimental evaluation of these compounds with known entry inhibitors.

## Figures and Tables

**Table 1:** Summary of natural product compound libraries used for virtual screening.

S/N	Company	Library	Number of Distinct Compounds
1	AnalytiCon Discovery	Fragment from nature (FRGx)	247
2		Macrocyclic semi-synthetic compounds (MACROx)	2040
3		Purified natural product screening compounds (MEGx)	3781
4	SPECS	Pre-plated 20k diverse compound library	20,636
5		Pre-plated natural product library	373
6	InterBioScreen (IBS)	IBS 2017_sep_Natual compound Libraries	747
Total			27,824

**Table 2:** Binding energies and interacting amino acid residues of recombinant clade A/E and clade B HIV-1 gp120.

	Recombinant Clade A/E		Clade B	
Compound	Binding Energy (kcal/mol)	Amino Acid Residues	Binding Energy (kcal/mol)	Amino Acid Residues
NP-008297	−10.3	Glu370, Gln258, Pro470, Ile371, Pro363, Ser365, Gln362, Gly471, Thr455, Gly472, and Asn425.	−7.9	Asp368, Ser365, Ser364, Gly366, Asn425, and Met426.
NP-000088	−9.7	Glu370, His375, Gln258, Gly472, Gln363, and Ser365	−7.1	Gly473, Thr283, Ser364, and Ser365.
NP-007382	−9.6	Pro470, Gly366, Ile371, Gly473, and Ile475.	−7.2	Asp457, Arg469, Ser364, Ser365, and Gly472.
NP-005003	−9.3	Asp457, Asn280, Pro470, and Arg469.	−7.3	Thr283 and Ala281.
NP-007422	−9.3	Gln258, Ile371, Thr257, Met373, Glu370, Asn425, Asp280, and Asp457.	−6.7	Asp474, Arg476, Trp427, Gly473, Asn425, Asp368, and Glu370.
NP-001800	−9.2	Glu370, Trp427, Gly429, Gln105, and Asn474.	−7.8	Ser365 and Gln363.
NP-005114	−9.1	Gln258, Ile371, Gln362, Pro363, Gly471, Asp457, Asn280, and Gly472.	−8.2	Arg476, Gly472, Pro470, Gly366, Ser364, Glu370, Asn425, Trp427, and Met426.
NP-004255	−9	Trp427, Gly429, Ile371, Gln258, Pro470, and Gly472.	−7.3	Arg476, Asp474, Thr455, and Pro470.
FRG-00075	−7.4	Glu370, Ile371, Ser365, Arg469, and Gly472.	−6.8	Gly431 and Ser375.

**Table 3:** Chemical structure of the nine selected compounds.

Compound ID	Chemical Structure
NP-000088	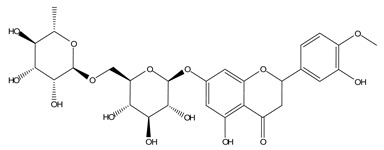
NP-004255	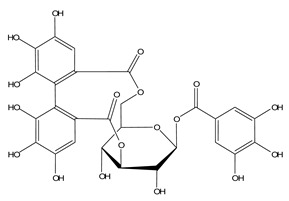
NP-005003	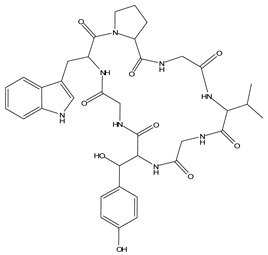
NP-005114	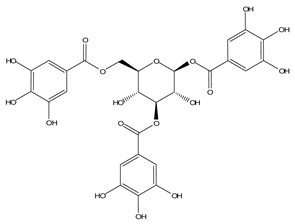
NP-007382	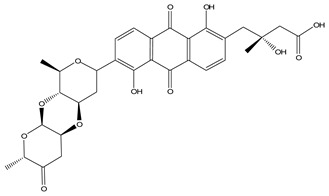
NP-007422	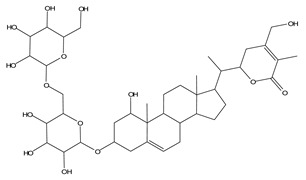
NP-008297	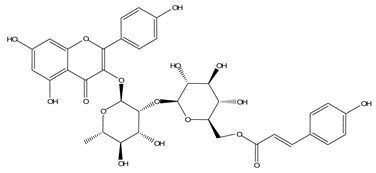
NP-001800	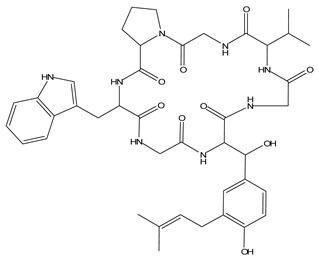
FRG-00075	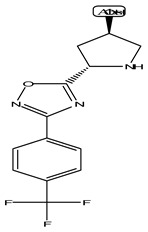

**Table 4:** The origin and sources of the test compounds. Purity data are as provided by the manufacturer.

Compound Structure	Organism	Name	Purity
NP-000088	Plant	*Mentha piperita*	79
NP-004255	Plant	*Terminalia chebula*	92
NP-005003	Micro-organism	*Aspergillus*	97
NP-005114	Plant	*Terminalia chebula*	99
NP-007382	Micro-organism	*Actinomycete*	98
NP-007422	Plant	*Withania somnifera*	96
NP-008297	Plant	*Ginkgo biloba*	94
NP-001800	Micro-organism	*Fungi*	79
FRG-00075	Fragment	*N/A*	70

**Table 5:** Physicochemical properties of the 9 selected compounds.

Compound Name	Molecular Weight (Dalton)	Number of Rotatable Bonds	Number of H-bond Acceptors	Number of H-bond Donors
NP-008297	740.66	9	16	9
NP-004255	634.45	2	15	11
NP-000088	610.56	7	15	8
NP-007422	782.91	9	14	9
NP-005114	636.46	5	15	11
NP-007382	596.57	5	12	4
NP-005003	723.78	5	9	9
NP-001800	800.90	7	9	9
FRG-00075	335.72	2	4	2

**Table 6:** Adsorption, distribution, metabolism, and excretion properties of the 9 selected compounds.

Compound Name	TPSA *	Lipophilicity	Water Solubility	GI Absorption ^¥^	BBB Permeant ^£^	Pgp Substrate ^§^	CYP Inhibitor ^ȡ^
NP-008297	275.5	1.79	Soluble	Low	No	Yes	No
NP-004255	310.66	−0.87	Soluble	Low	No	Yes	No
NP-000088	234.29	−0.43	Soluble	Low	No	Yes	No
NP-007422	245.29	0.07	Soluble	Low	No	Yes	No
NP-005114	310.66	−3.12	Soluble	Low	No	No	No
NP-007382	186.12	1.91	Soluble	Low	No	Yes	No
NP-005003	251.16	−0.98	Soluble	Low	No	Yes	No
NP-001800	251.16	0.61	Insoluble	Low	No	Yes	No
FRG-00075	71.18	1.3	Soluble	High	No	Yes	No

* Topological polar surface area. § P-glycoprotein substrate. ¥ gut absorption. £ cross the blood–brain barrier. ȡ CYP450 conversion.

## Data Availability

Not applicable.

## Supplementary Materials

The following supporting information can be downloaded at: https://www.mdpi.com/article/10.3390/molecules28020474/s1, Figure S1: A typical 96-well plate layout for Alamar blue cell viability assay; Figure S2: Viral infectivity inhibition assay plate layout; Figure S3: Compounds A) NP-008297 and B) NP-007382 docked into CD4-bs (hot pink shaded) of recombinant clade A/E HIV gp120 (blue molecular surface); Figure S4: Protein–ligand interaction profiles of recombinant clade A/E HIV gp120- A) NP-008297 and B) NP-007382 complexes; Figure S5: Summary of virtual high-throughput screening. The best 600 ligands were selected from the ligand library based on binding energies, and 470 ligands of the selected ligands were analyzed. A total of 304 ligands had hydrogen bond interactions with the CD4 binding site (CD4-bs) amino acid residues. Forty-one had hydrogen bond interactions with 4 or more amino acid residues in the CD4-bs. The best 10 compounds were selected from the 41 for in vitro analysis; Figure S6: Determination of the 3D structure of HIV HXB2. A) The amino acid sequence of the HXB2 envelope protein retrieved from the HIV database, B) BlastP similarity search result of HXB2 env amino acid sequence (sequence alignment), C) 3D structure of 3J70, which consists of HXB2 gp160 in complex with CD4 and 17b antibody, and D) HXB2 gp120 extracted from 3J70 using PyMOL; Figure S7: Molecular interactions between the CD4-bs of clade B HIV gp120 (blue molecular surface) and compounds NP-008297 (A and B) and NP-007382 (C and D); Figure S8: RMSD analysis of MD simulation trajectory. The plots for A) HXB2-NP-005114, B) HXB2-NP-007382, C) HXB2-NP-007422, and D) HXB2-NP-008297 complexes; Figure S9: RMSF analysis of MD simulation trajectory. The plots for A) clade A/E-NP-005114, B) clade A/E-NP-007382, C) clade A/E-NP-007422, and D) clade A/E-NP-008297 complexes. For the RMSF plots, the residue index 1 maps to Val44; Figure S10: RMSF analysis of MD simulation trajectory. The plots for A) HXB2-NP-005114, B) HXB2-NP-007382, C) HXB2-NP-007422, and D) HXB2-NP-008297 complexes; Figure S11: Analysis of the molecular interactions and the type of contacts with HXB2 throughout MD simulation. Normalized stacked bar chart of HXB2 residues interacting with A) NP-005114, B) NP-007382, C) NP-007422, and D) NP-008297. Hydrogen bond, hydrophobic bond, ionic interactions, and water bridges are represented as green, grey, red, and blue, respectively; Figure S12: Alamar blue cell viability assay shows the tested compounds had no cytotoxic effect on the TZM-bl cells. The positive control, ursolic acid, is a known cytotoxic compound. Error bars represent the standard error of the mean (SEM) of triplicate wells; Figure S13: Determination of 50% cytotoxicity concentration (CC_50._). Alamar blue cell viability assay shows NP-004255 and NP-007382 had a cytotoxic effect on the TZM-bl cells (A and B). The positive control, ursolic acid, is a known cytotoxic compound. CC_50_ was determined using dose–response non-linear regression analysis. (C) NP-004255 with CC_50_ of 14.4 µM (9.1 µg/mL) and (D) NP-007382 with CC_50_ of 47.6 µM (28.4 µg/mL). Error bars represent the standard error of the mean (SEM) of triplicate wells; Figure S14: Viral infectivity inhibition assay for A) FRG-00075, B) NP-000088, C) NP-001800, D) NP-004255, and E) NP-005003. A dose-independent inhibitory activity was observed for NP-004255. No inhibition was observed for compounds FRG-00075, NP-0005003, NP-000088, and NP-001800. Error bars represent standard error of the mean (SEM); Table S1: Maximum concentration of test compound used for cell viability and viral inhibition; Table S2: Toxicity profiles of the 9 selected compounds predicted using ProTox-II.

## Sample Availability

Samples of the compounds are available from the authors.
