# Supplementary material for: Structure-Based Identification of Natural-Product-Derived Compounds with Potential to Inhibit HIV-1 Entry
Source: Molecules. 2023 Jan 4;28(2):474. doi: 10.3390/molecules28020474 (PMC9865492; doi:10.3390/molecules28020474)
Supplement: Supplementary file 1 [file molecules-28-00474-s001.zip › molecules-1924649-supplementary.pdf]

Supplementary Tables

Table S1. Maximum concentration of test compound used for cell viability and viral inhibition.

Compound	Maximum Assay Concentration	
	Cell Viability Assay	Viral Infectivity Assay
NP-000088	164 µM (100 µg/ml)	164 µM (100 µg/ml)
NP-004255	158 µM (100 µg/ml)	158 µM (100 µg/ml)
NP-005003	136 µM (100 µg/ml)	136 µM (100 µg/ml)
NP-005114	157 µM (100 µg/ml)	157 µM (100 µg/ml)
NP-007382	168 µM (100 µg/ml)	42 µM (25 µg/ml)
NP-007422	128 µM (100 µg/ml)	128 µM (100 µg/ml)
NP-008297	135 µM (100 µg/ml)	135 µM (100 µg/ml)
NP-001800	125 µM (100 µg/ml)	125 µM (100 µg/ml)
FRG-00075	334 µM (100 µg/ml)	334 µM (100 µg/ml)

Table S2. Toxicity profiles of the 9 selected compounds predicted using ProTox-II.

Compound Name	Toxicity Class	Hepatotoxicity	Carcinogenicity	Immunotoxicity	Mutagenicity	Cytotoxicity	LD_{50} (mg/kg)
NP-008297	4	Inactive	Inactive	Active	Inactive	Inactive	1000
NP-004255	5	Inactive	Inactive	Active	Inactive	Inactive	2260
NP-000088	6	Inactive	Inactive	Active	Inactive	Inactive	5530
NP-007422	2	Inactive	Inactive	Active	Inactive	Active	7
NP-005114	6	Inactive	Inactive	Inactive	Inactive	Inactive	8000
NP-007382	4	Inactive	Inactive	Active	Inactive	Inactive	2000
NP-005003	4	Inactive	Inactive	Active	Inactive	Inactive	500
NP-001800	4	Inactive	Inactive	Active	Inactive	Inactive	1000
FRG-00075	4	Inactive	Active	Inactive	Inactive	Inactive	1000

Supplementary Figures

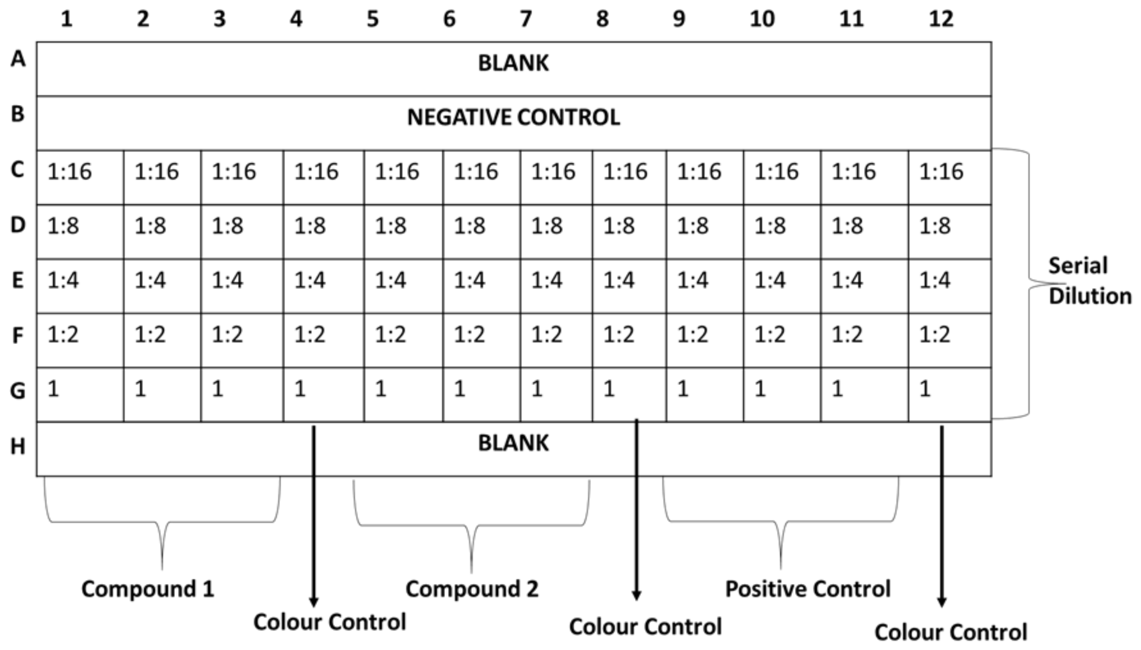

Figure S1. A typical 96-well plate layout for Alamar blue cell viability assay.

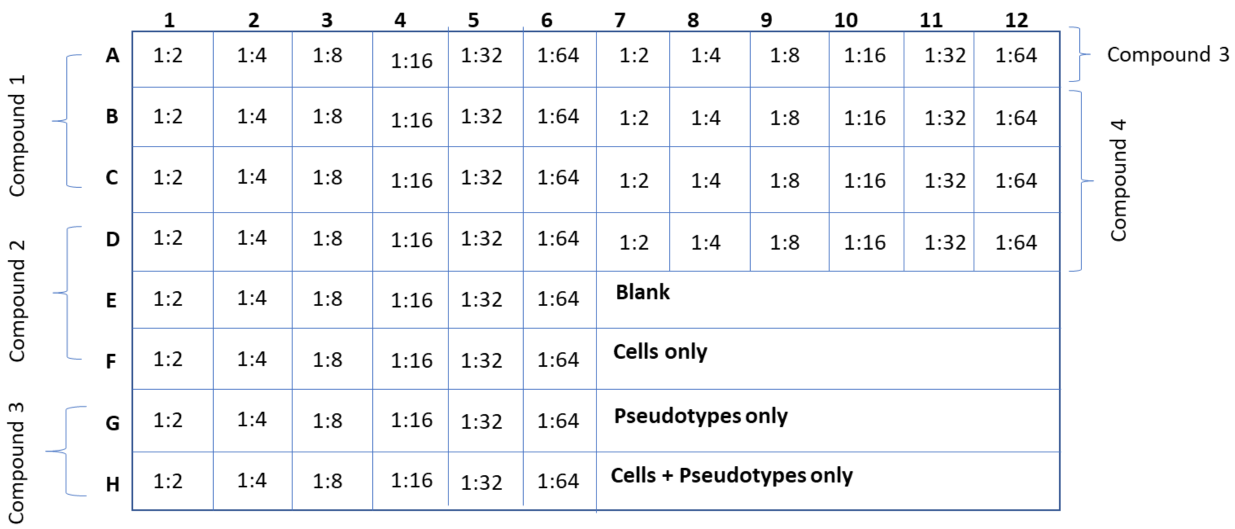

Figure S2. Viral infectivity inhibition assay plate layout.

A)

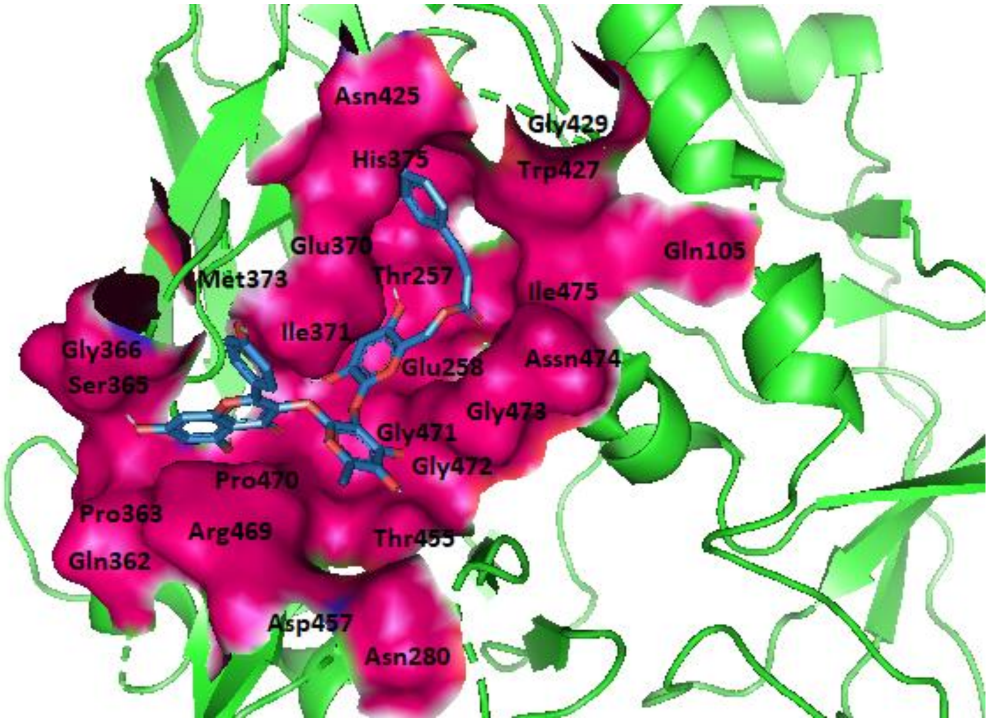

B)

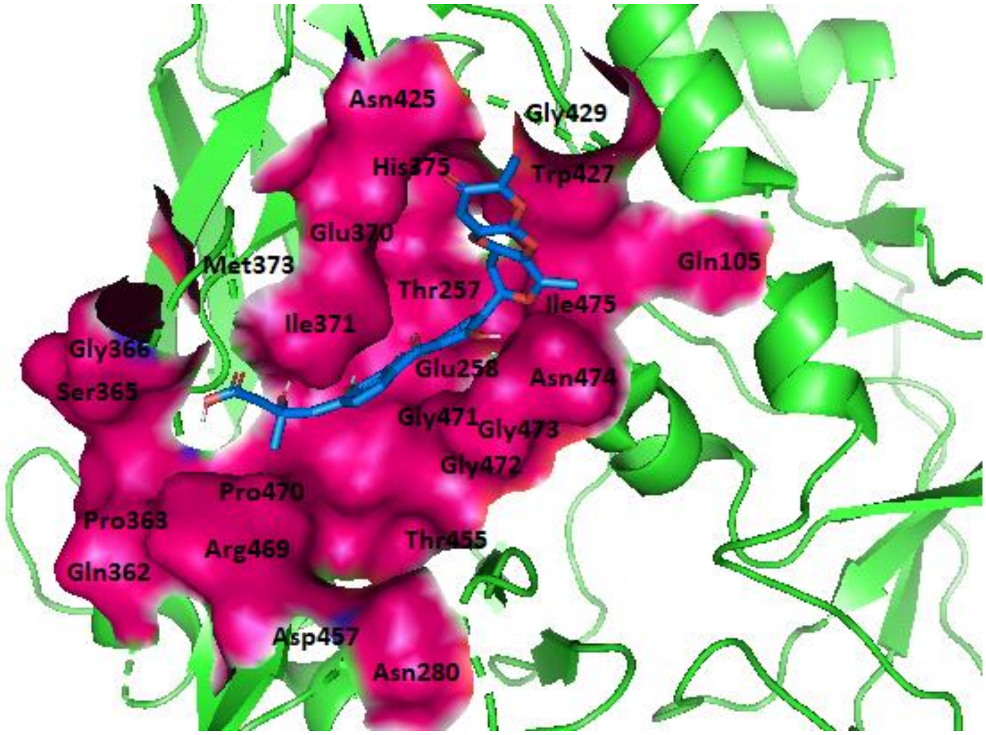

Figure S3. Compounds A) NP-008297 and B) NP-007382 docked into CD4-bs (hotpink shaded) of recombinant clade A/E HIV-gp120 (blue molecular surface).

A)

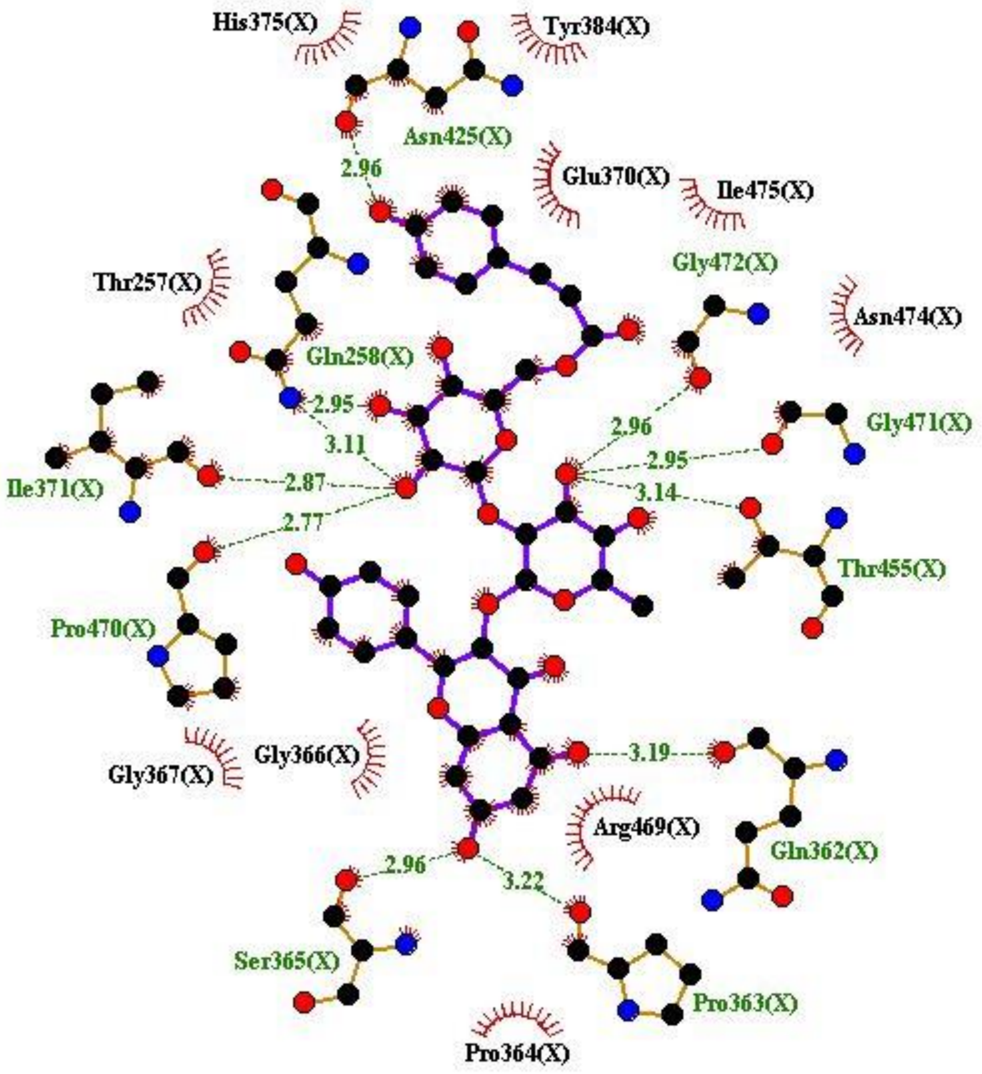

His375(X)
Tyr384(X)
Asn425(X)
2.96
Glu370(X)
Ile475(X)
Thr257(X)
Gly472(X)
Asn474(X)
Gln258(X)
2.95
3.11
2.96
Gly471(X)
2.95
Ile371(X)
2.87
3.14
2.77
Thr455(X)
Pro470(X)
Gly367(X)
Gly366(X)
3.19
Arg469(X)
Gln362(X)
2.96
3.22
Ser365(X)
Pro363(X)
Pro364(X)

B)

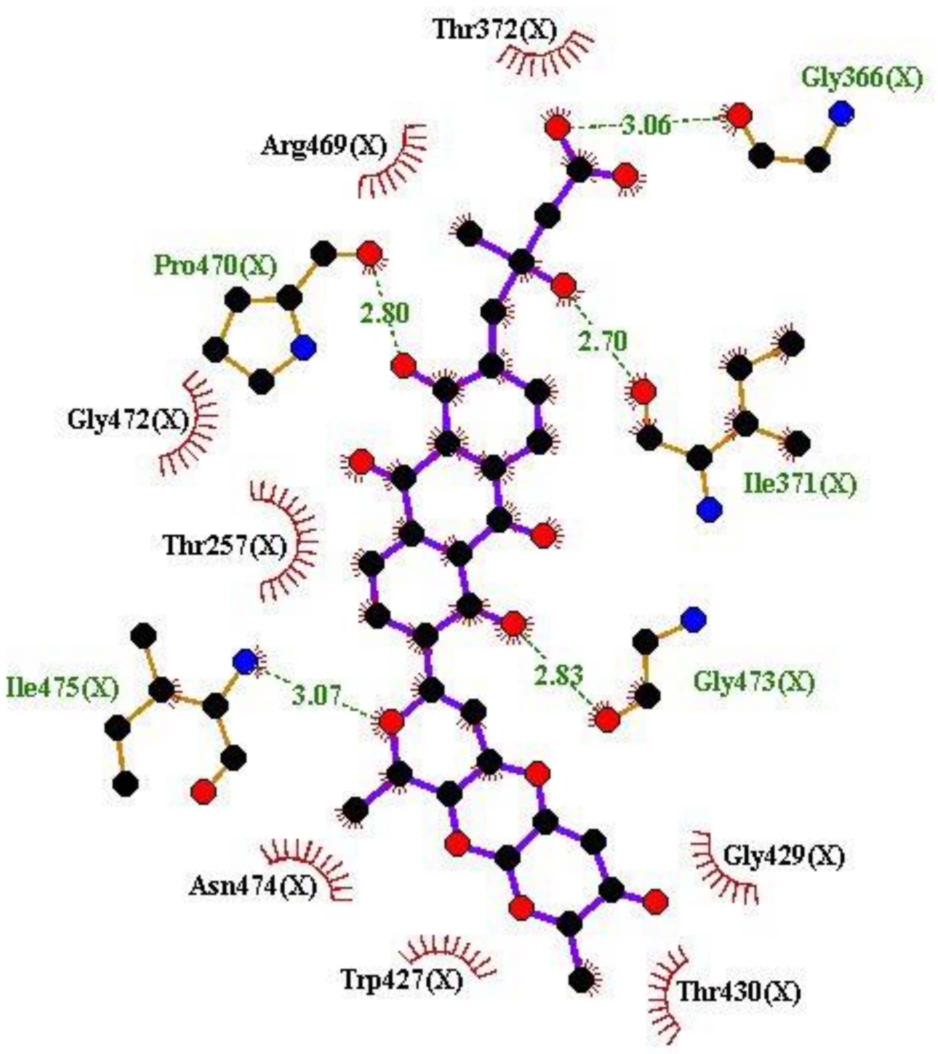

Figure S4. Protein-ligand interaction profiles of recombinant clade A/E HIV gp120- A) NP-008297 and B) NP-007382 complexes.

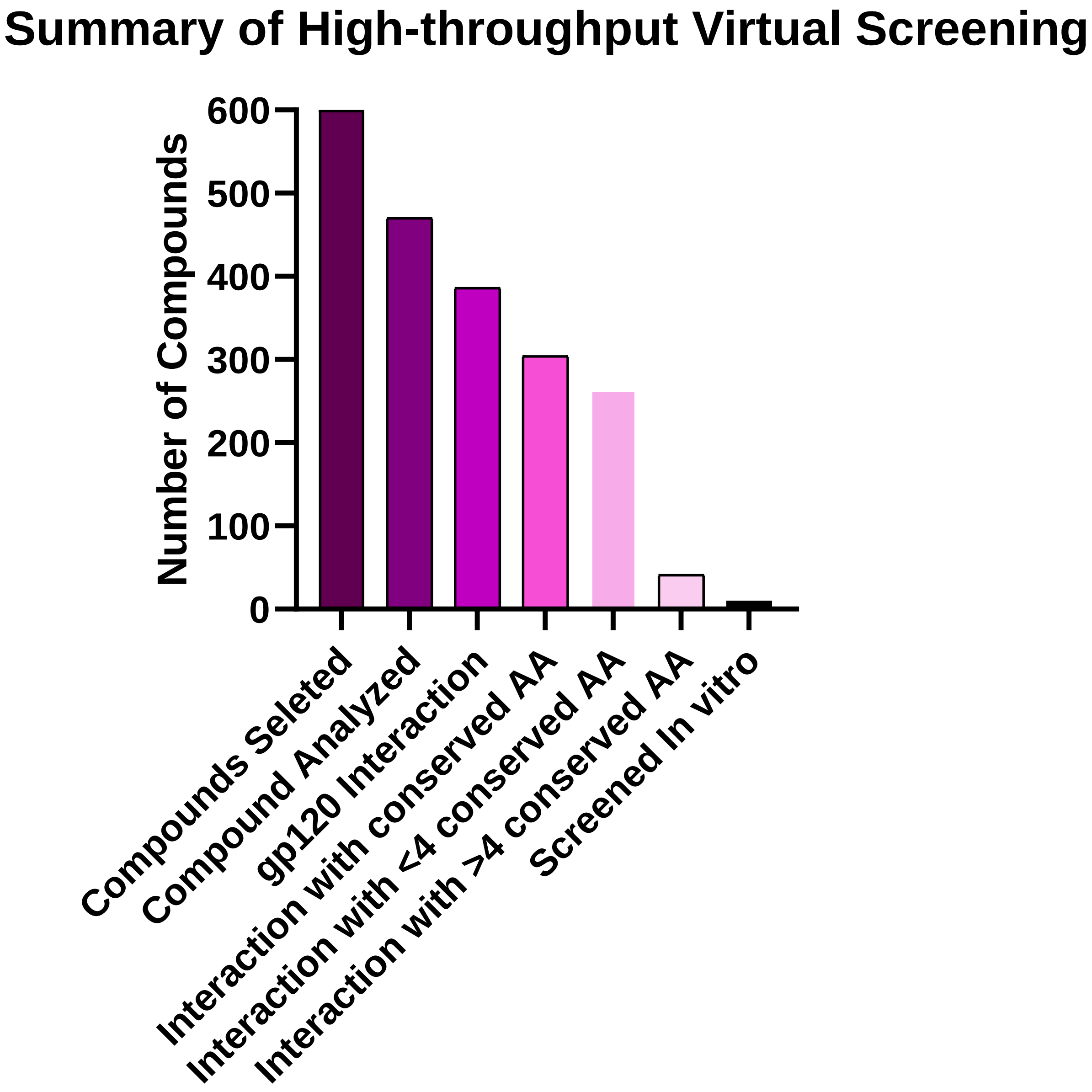

Figure S5. Summary of virtual high throughput screening. The best 600 ligands were selected from the ligand library based on binding energies and 470 ligands of the selected ligands were analysed. A total of 304 ligands had hydrogen bond interactions with the CD4-Binding site (CD4-bs) amino acid residues. 41 had hydrogen bond interactions with 4 or more amino acid residues in the CD4-bs. The best 10 compounds were selected from the 41 for in vitro analysis.

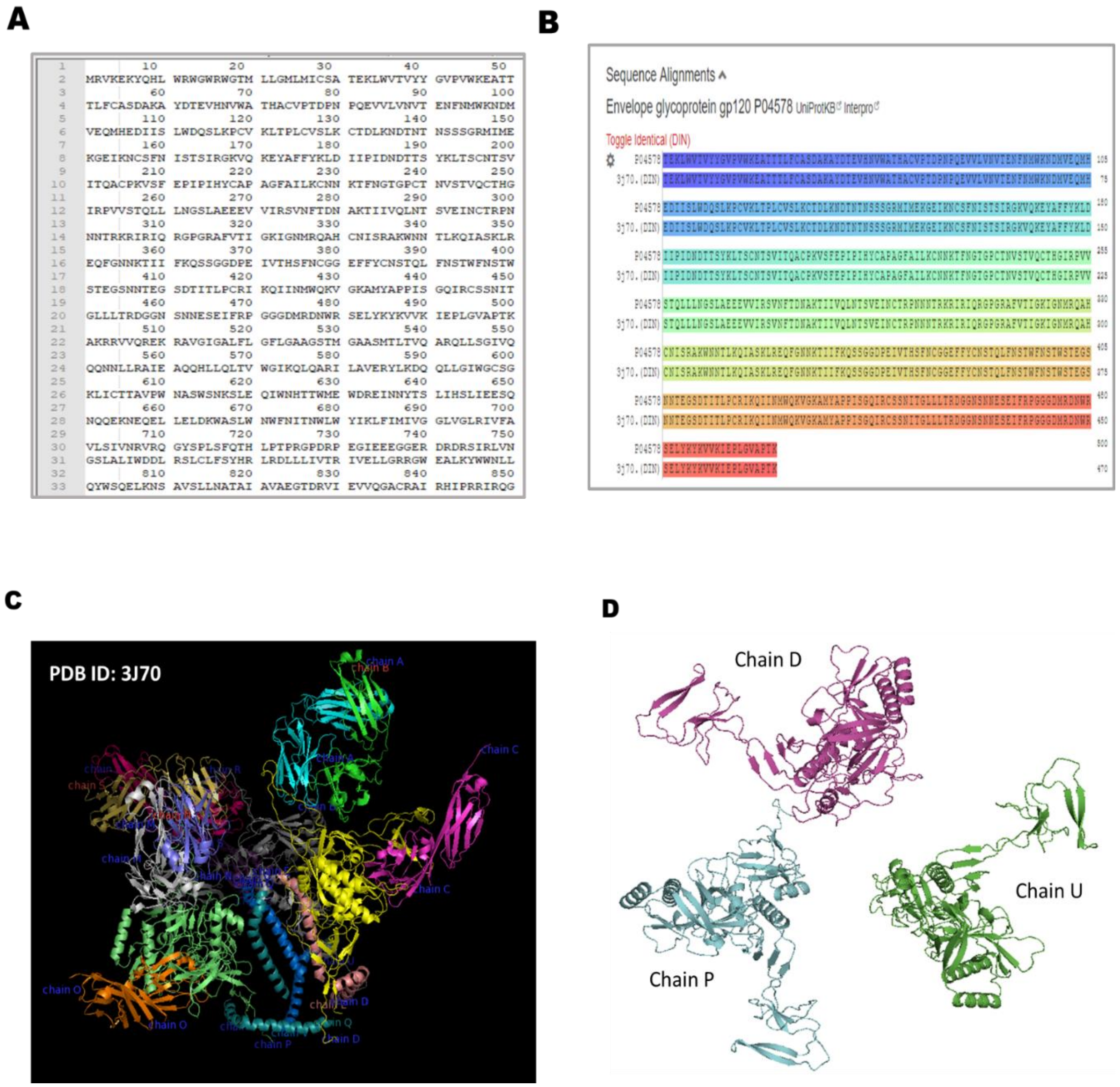

Figure S6. Determination of the 3-D structure of HIV HXB2. A) The amino acid sequence of the HXB2 envelope protein retrieved from the HIV database, B) BlastP similarity search result of HXB2 Env amino acid sequence (sequence alignment), C) 3-D structure of 3J70, which consist of HXB2 gp160 in complex with CD4 and 17b antibody, and D) HXB2 gp120 extracted from 3J70 using PyMOL.

A)

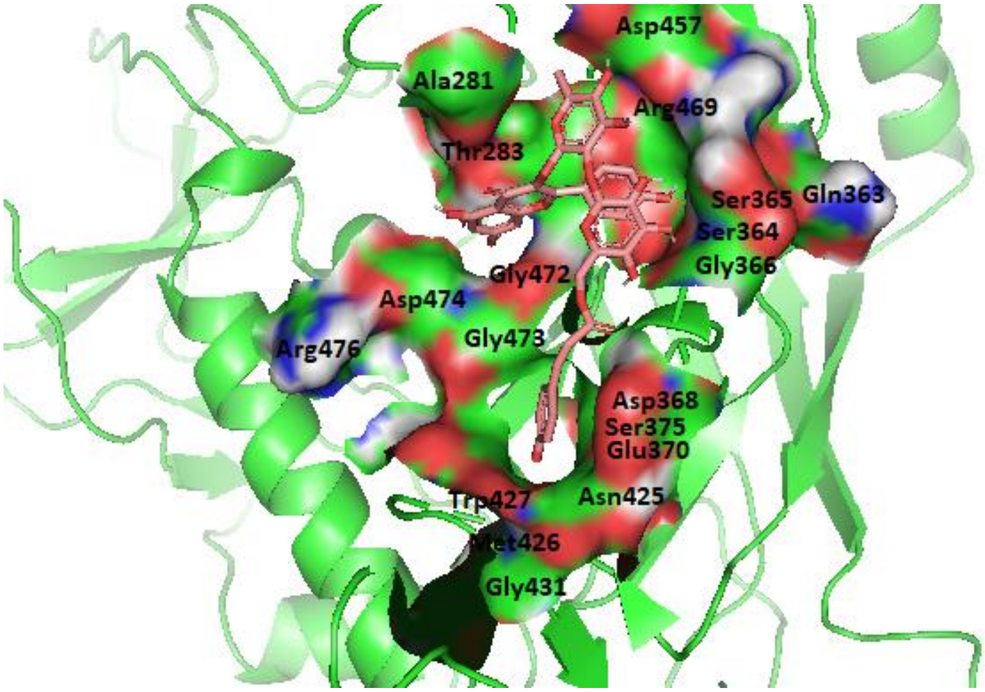

Asp457
Ala281
Arg469
Thr283
Ser365
Gln363
Ser364
Gly366
Gly472
Asp474
Arg476
Gly473
Asp368
Ser375
Glu370
Trp427
Asn425
Met426
Gly431

B)

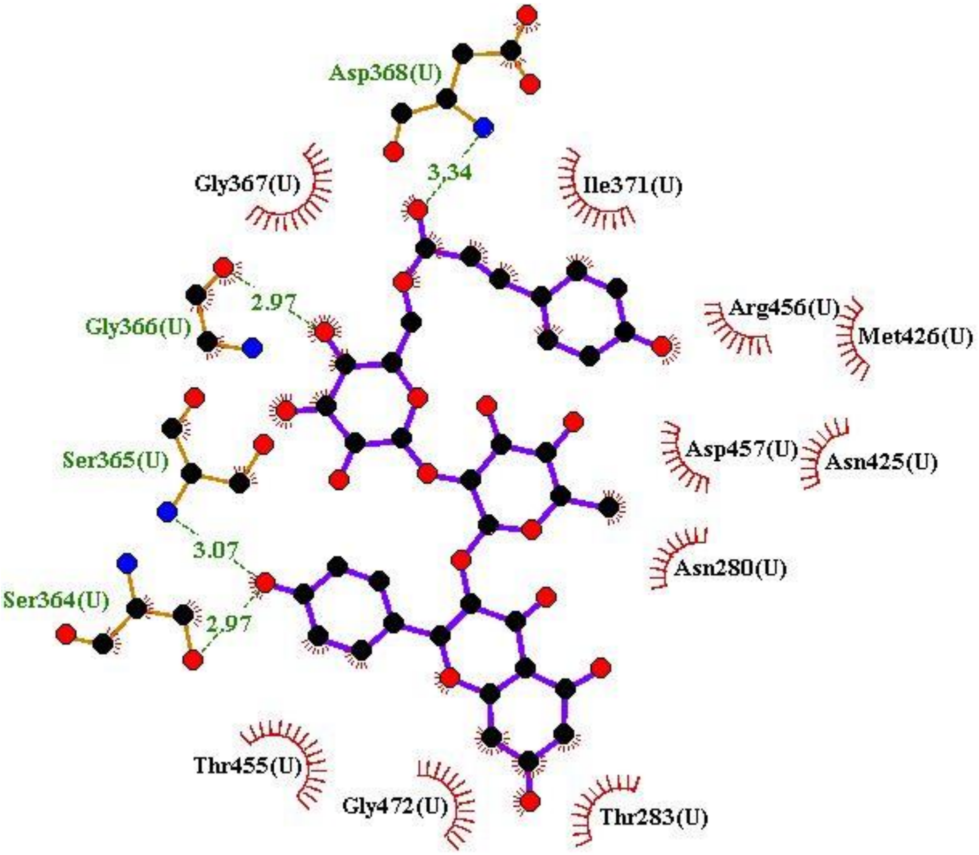

Asp368(U)
3.34
Gly367(U)
Ile371(U)
2.97
Gly366(U)
Arg456(U)
Met426(U)
Ser365(U)
Asp457(U)
Asn425(U)
3.07
Asn280(U)
Ser364(U)
2.97
Thr455(U)
Gly472(U)
Thr283(U)

C)

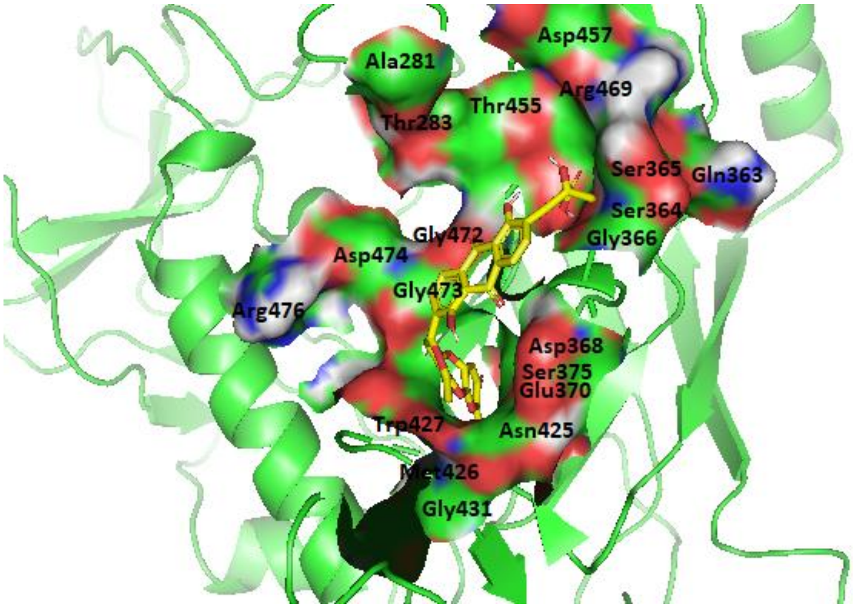

D)

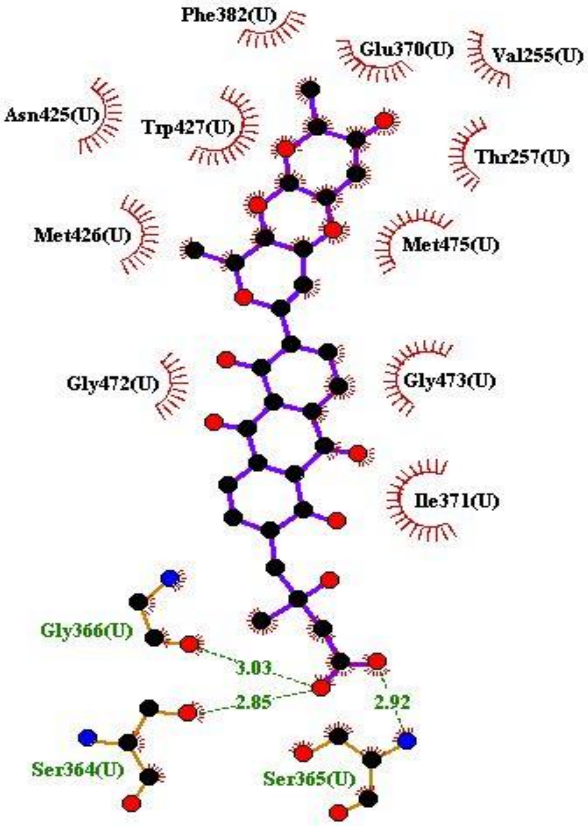

Figure S1. Molecular interactions between the CD4-bs of clade B HIV-gp120 and compounds NP-008297 (A and B) and NP-007382 (C and D).

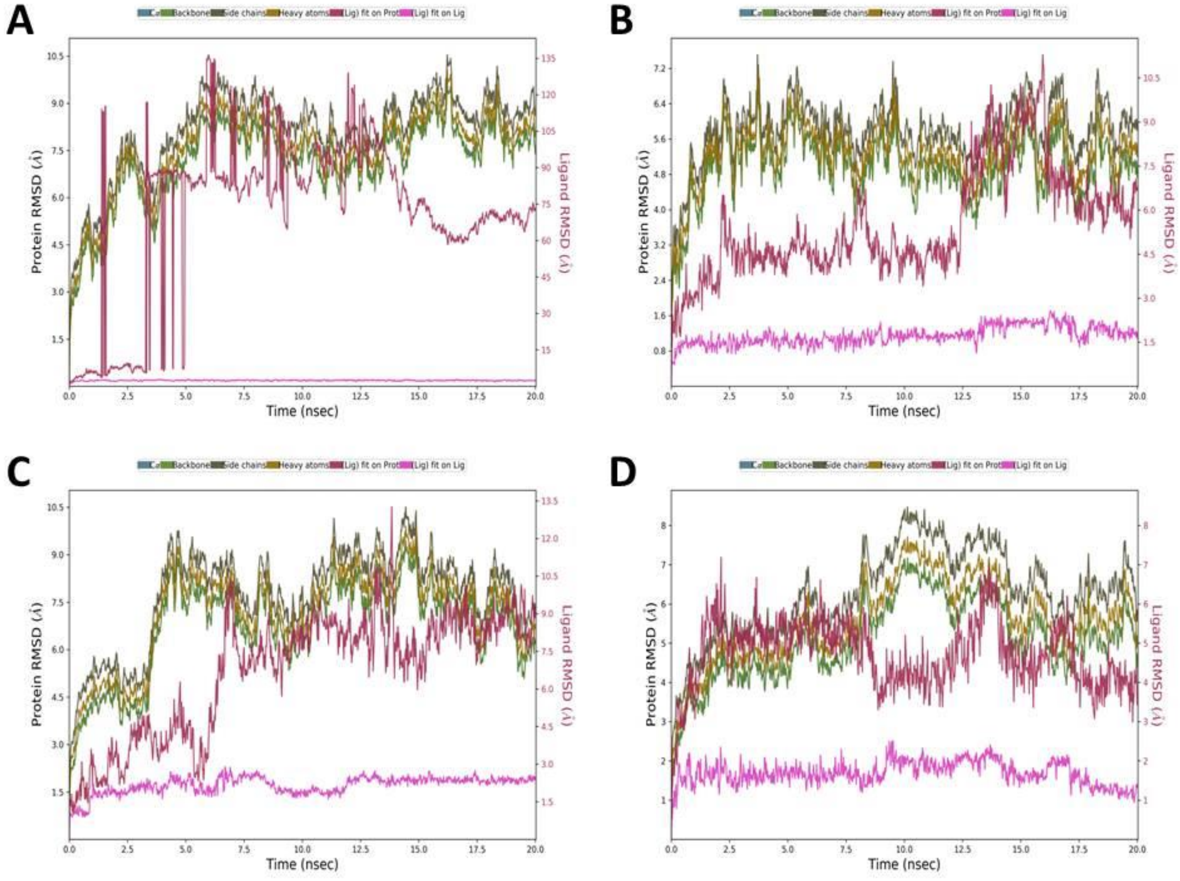

Figure S8. RMSD analysis of MD simulation trajectory. The plots for A) HXB2-NP-005114, B) HXB2-NP-007382, C) HXB2-NP-007422 and D) HXB2-NP-008297 complexes.

A)

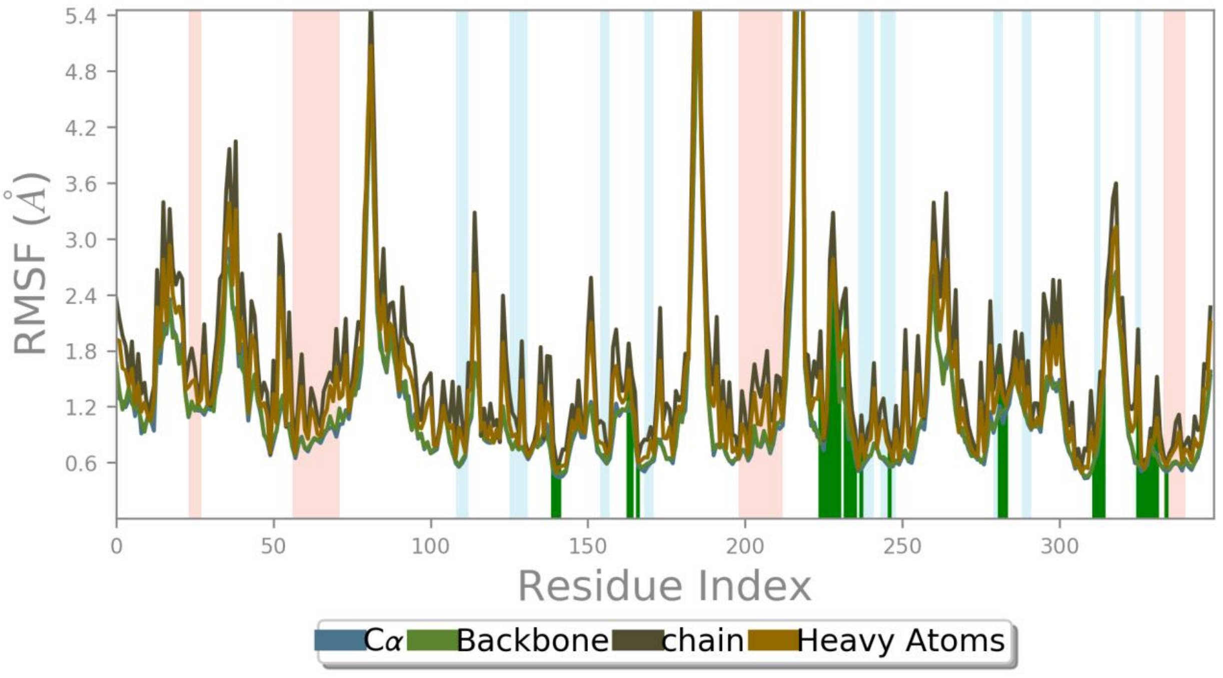

5.4
4.8
4.2
3.6
3.0
2.4
1.8
1.2
0.6
RMSF (Å)
0
50
100
150
200
250
300
Residue Index
Cα
Backbone
chain
Heavy Atoms

B)

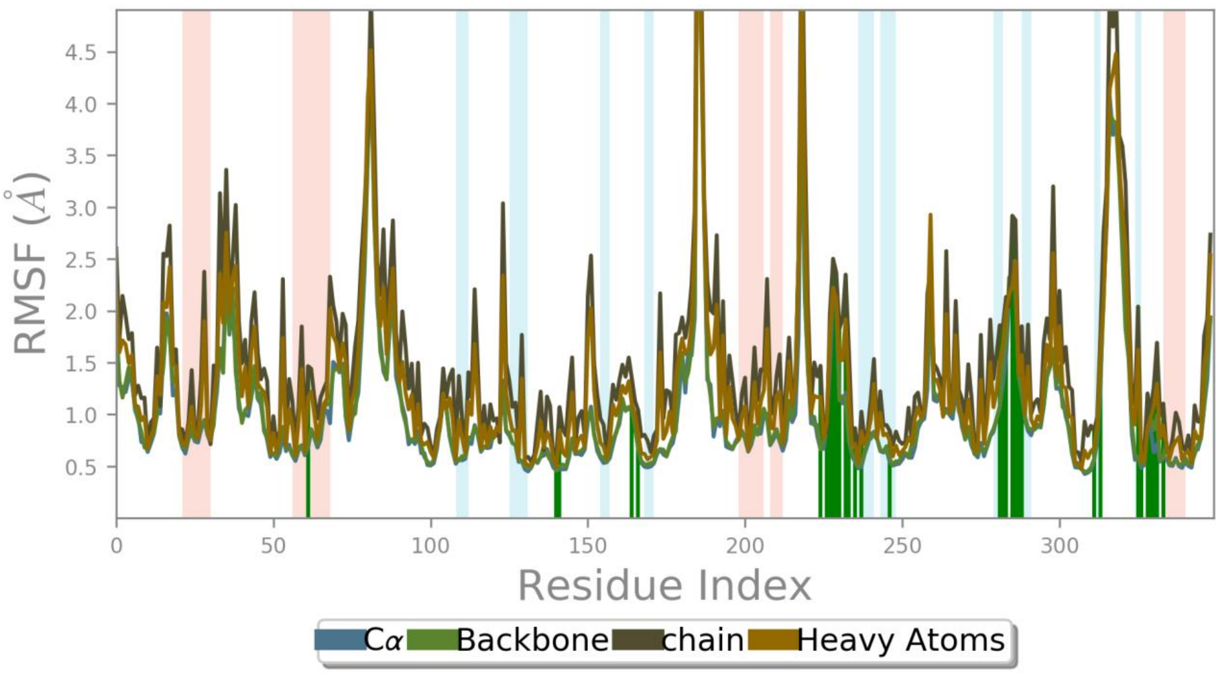

4.5
4.0
3.5
3.0
2.5
2.0
1.5
1.0
0.5
RMSF (Å)
0
50
100
150
200
250
300
Residue Index
Cα
Backbone
chain
Heavy Atoms

C)

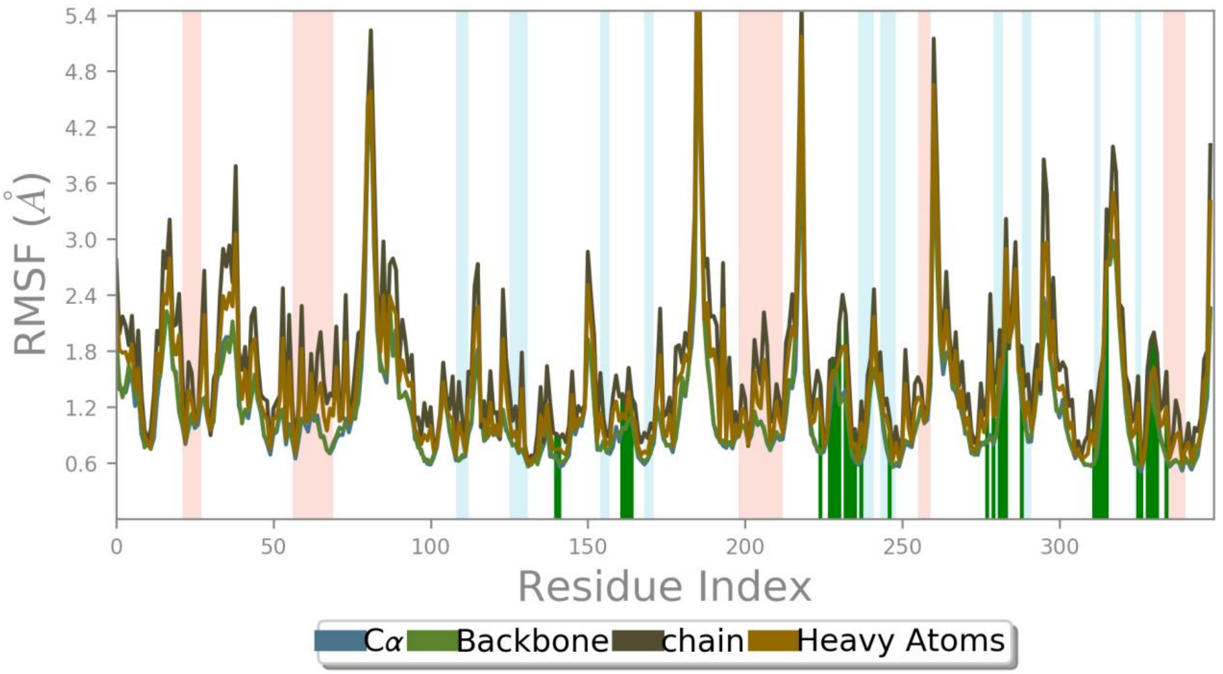

5.4
4.8
4.2
3.6
3.0
2.4
1.8
1.2
0.6
RMSF (Å)
0
50
100
150
200
250
300
Residue Index
Cα
Backbone
chain
Heavy Atoms

D)

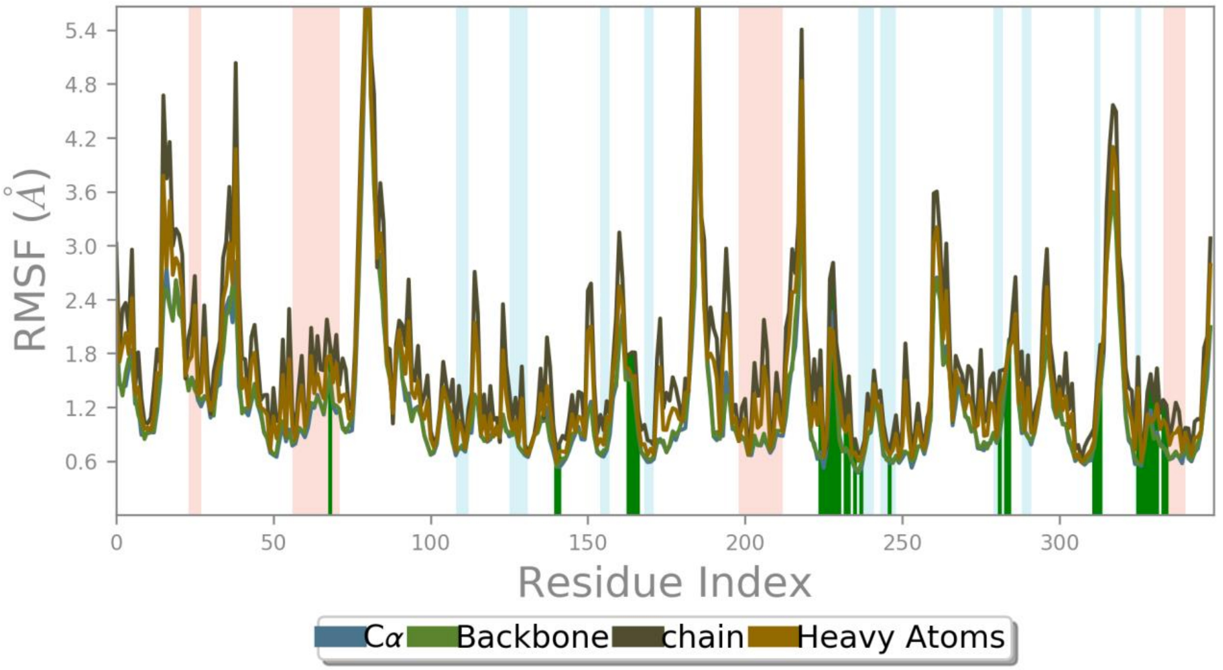

5.4
4.8
4.2
3.6
3.0
2.4
1.8
1.2
0.6
RMSF (Å)
0
50
100
150
200
250
300
Residue Index
Cα
Backbone
chain
Heavy Atoms

Figure S9. RMSF analysis of MD simulation trajectory. The plots for A) Clade A/E-NP-005114, B) Clade A/E-NP-007382, C) Clade A/E-NP-007422 and D) Clade A/E-NP-008297 complexes. For the RMSF plots, the residue index 1 maps to Val44.

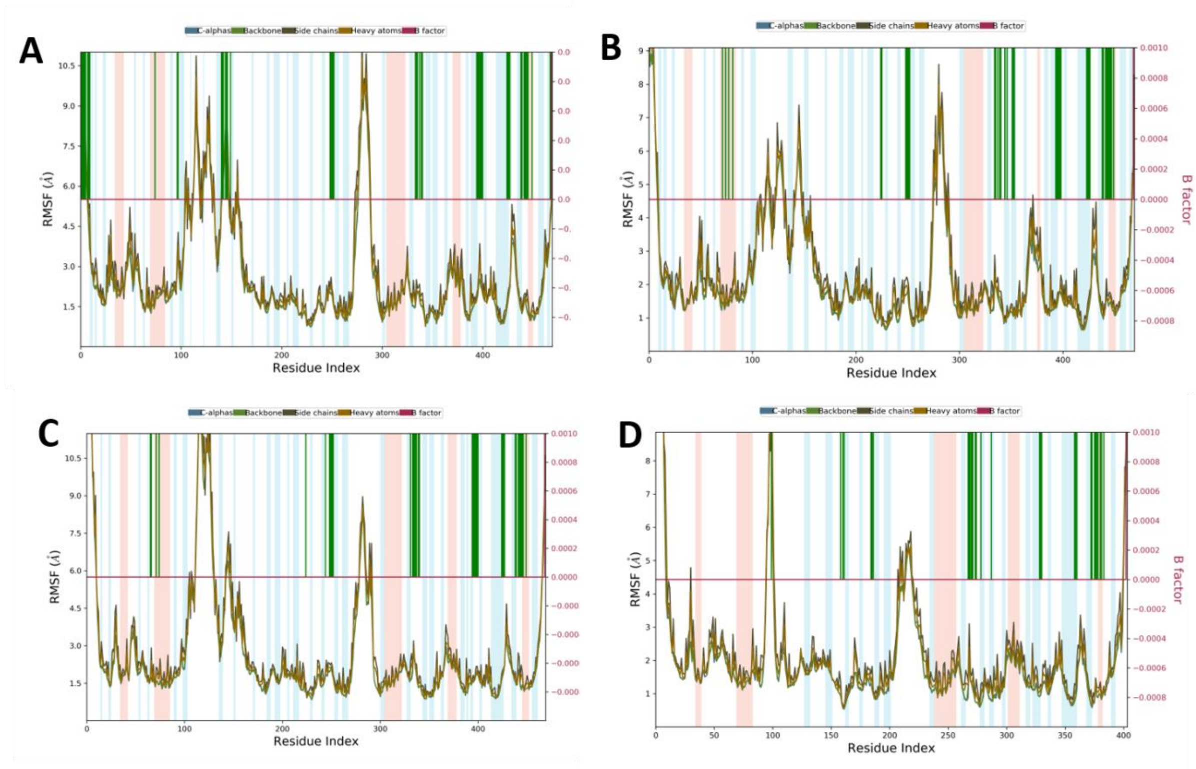

Figure S10. RMSF analysis of MD simulation trajectory. The plots for A) HXB2-NP-005114, B) HXB2-NP-007382, C) HXB2-NP-007422 and D) HXB2-NP-008297 complexes.

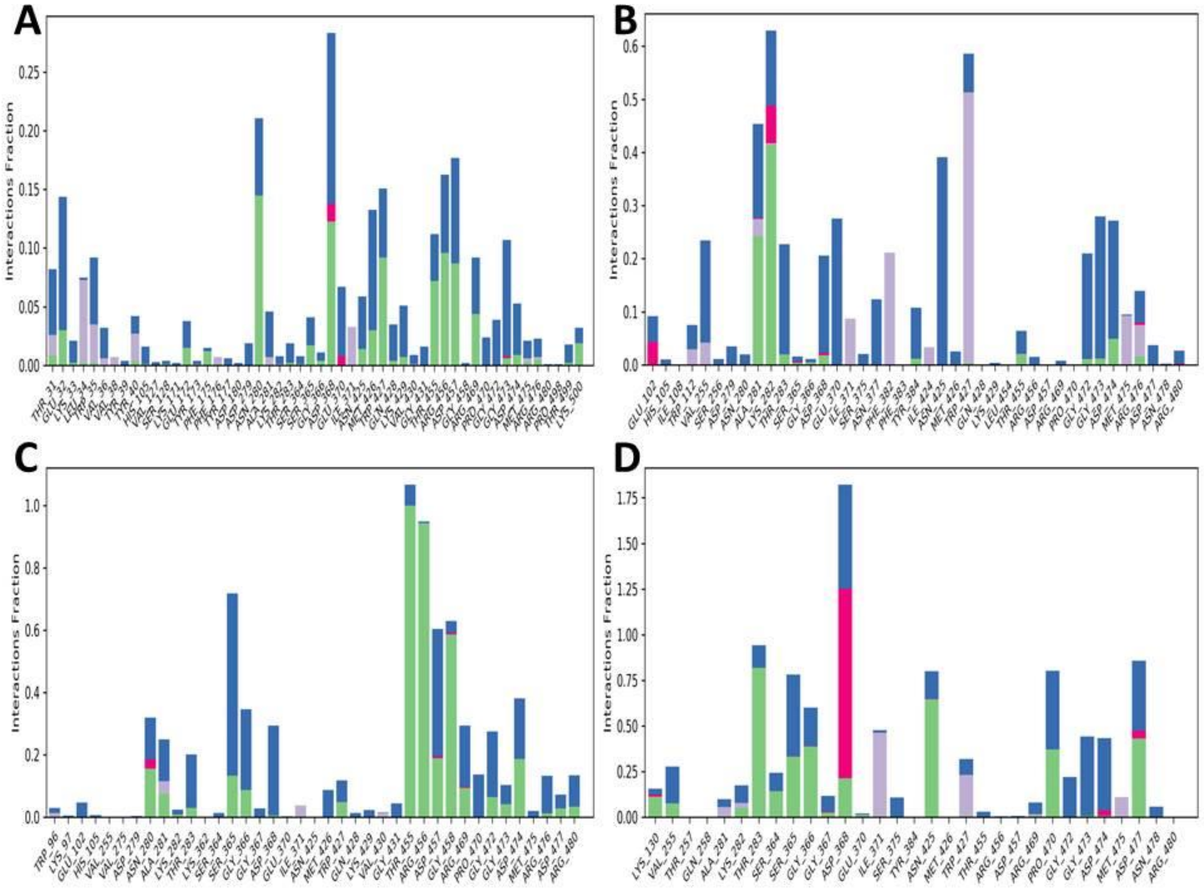

Figure S11. Analysis of the molecular interactions and the type of contacts with HXB2 throughout MD simulation. Normalized stacked bar chart of HXB2 residues interacting with A) NP-005114, B) NP-007382, C) NP-007422 and D) NP-008297. Hydrogen bond, hydrophobic bond, ionic interactions, and water bridges are represented as green, grey, red and blue, respectively.

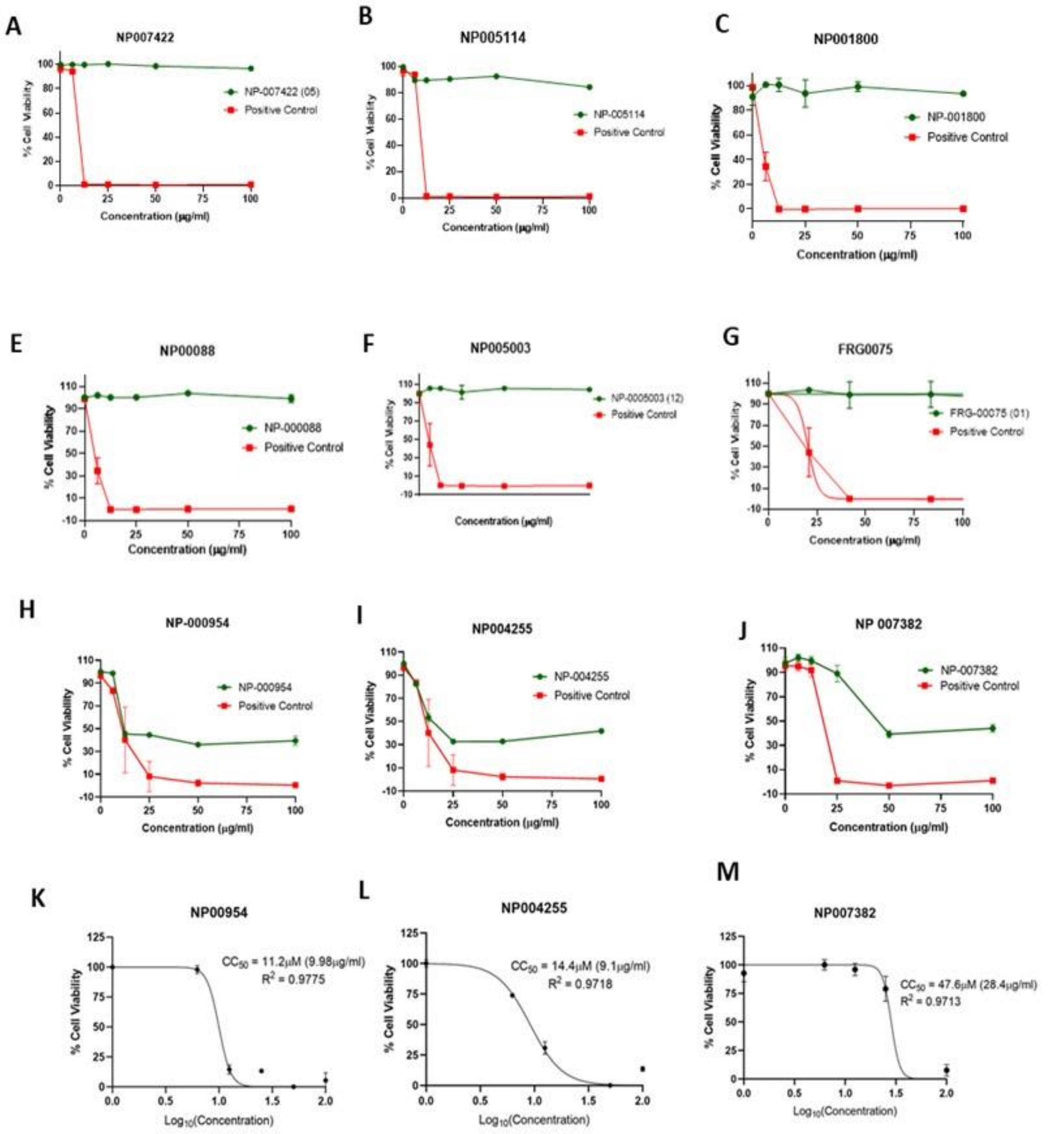

Figure S12. Alamar blue cell viability assay shows the tested compounds had no cytotoxic effect on the TZM-bl cells. The positive control, ursolic acid, is a known cytotoxic compound. Error bars represent the standard error of the mean (SEM) of triplicate wells.

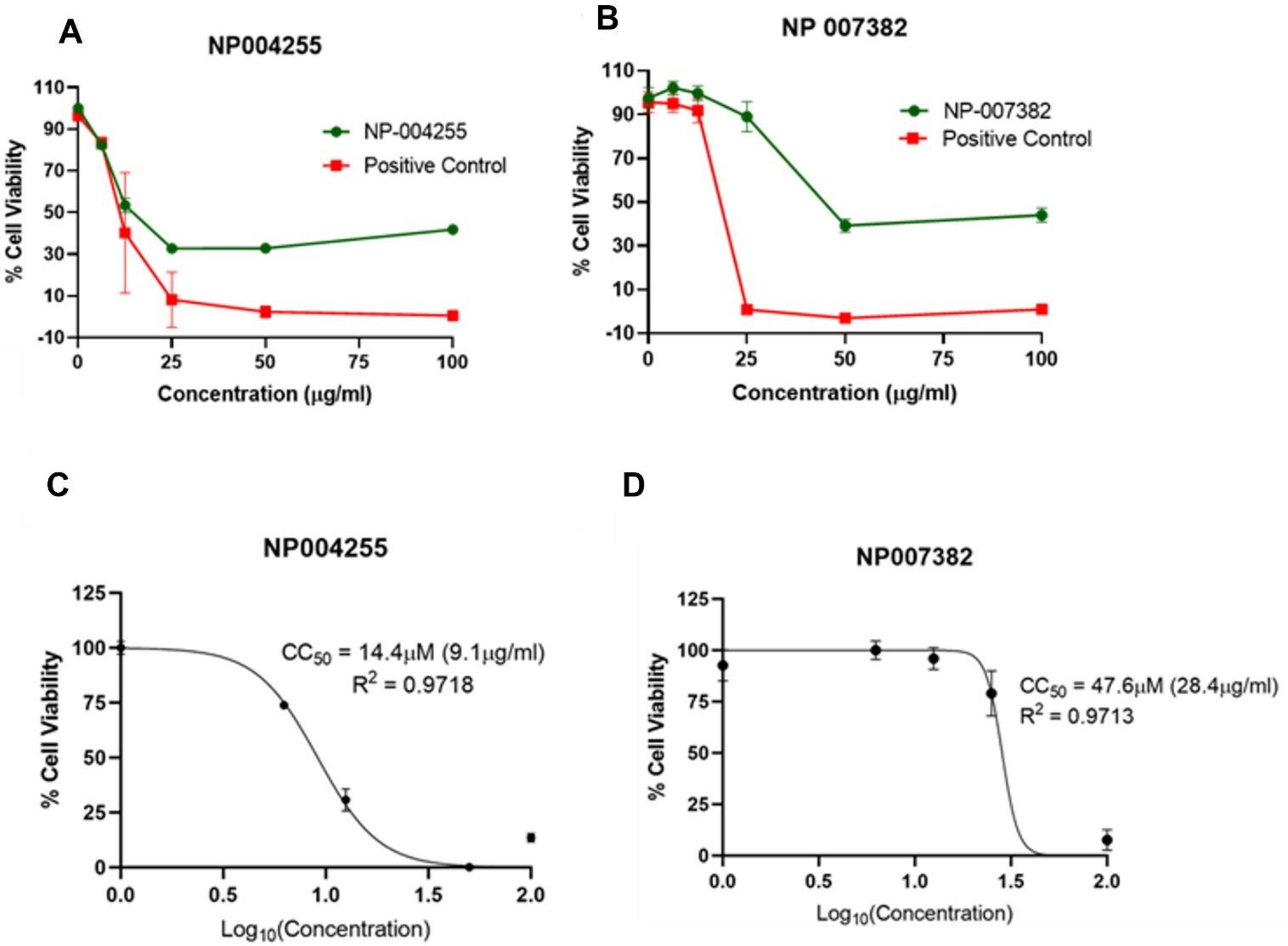

Figure S13. Determination of 50% cytotoxicity concentration ($CC_{50.}$). Alamar blue cell viability assay shows NP-004255 and NP-007382 had a cytotoxic effect on the TZM-bl cells (A and B). The positive control, ursolic acid, is a known cytotoxic compound. CC_{50} was determined using dose-response non-linear regression analysis. (C) NP-004255 with CC_{50} of 14.4 µM (9.1µg/ml) and (D) NP-007382 with CC_{50} of 47.6 µM (28.4µg/ml). Error bars represent the standard error of the mean (SEM) of triplicate wells.

A)

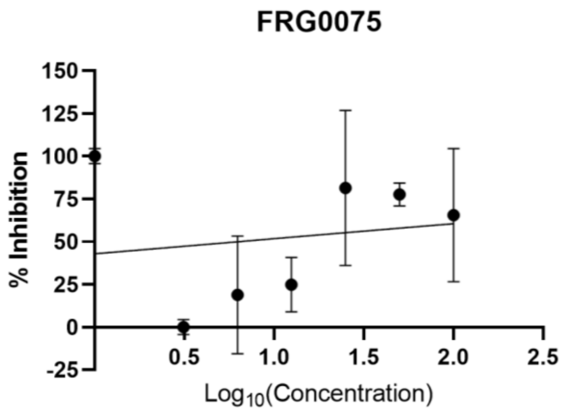
FRG0075
% Inhibition
Log10(Concentration)

B)

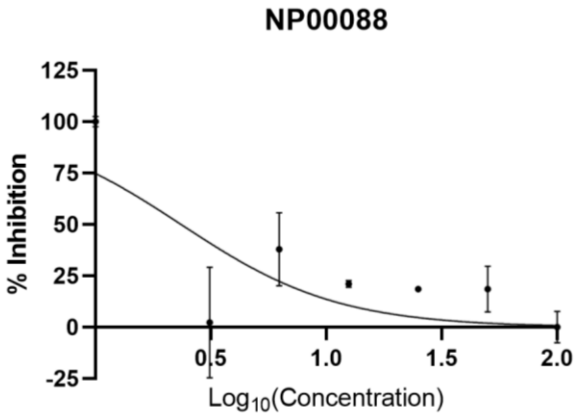
NP00088
% Inhibition
Log10(Concentration)

C)

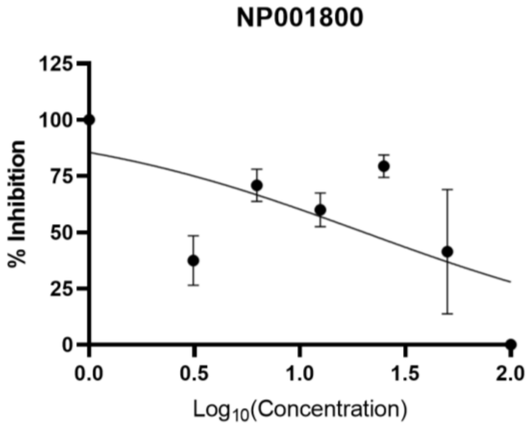
NP001800
% Inhibition
Log10(Concentration)

D)

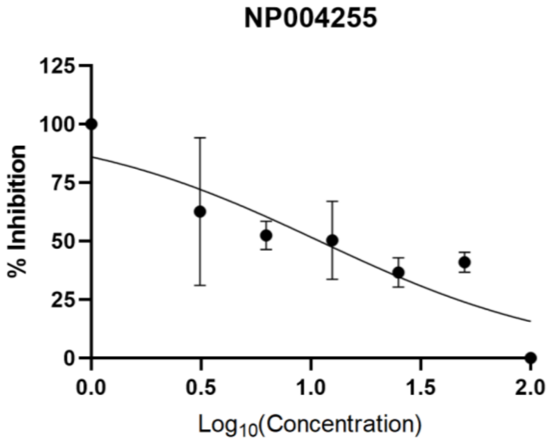

E)

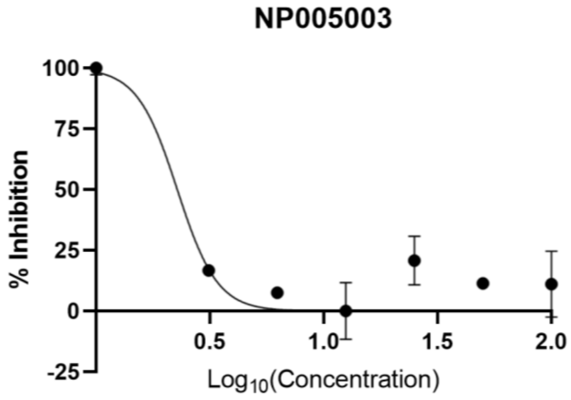

Figure S14. Viral infectivity inhibition assay for A) FRG-00075, B) NP-000088, C) NP-001800, D) NP-004255 and E) NP-005003. A dose-independent inhibitory activity was observed for NP-004255. No inhibition was observed for compounds FRG-00075, NP-0005003, NP-000088 and NP-001800. Error bars represent standard error of the mean (SEM).
